# Pauling-type adsorption of O_2_ induced electrocatalytic singlet oxygen production on N–CuO for organic pollutants degradation

**DOI:** 10.1038/s41467-022-33149-4

**Published:** 2022-09-22

**Authors:** Liangbo Xie, Pengfei Wang, Yi Li, Dongpeng Zhang, Denghui Shang, Wenwen Zheng, Yuguo Xia, Sihui Zhan, Wenping Hu

**Affiliations:** 1 Tianjin Key Laboratory of Molecular Optoelectronic Sciences, Department of Chemistry, School of Science, Tianjin University & Collaborative Innovation Center of Chemical Science and Engineering (Tianjin), Tianjin, 300072 China; 2 Tianjin Key Lab Clean Energy & Pollutant Control, School of Energy and Environmental Engineering, Hebei University of Technology, Tianjin, 300130 China; 3 Joint School of National University of Singapore and Tianjin University, Fuzhou International Campus, Tianjin University, Binhai New City, Fuzhou, 350207 China; 4 Key Laboratory of Pollution Processes and Environmental Criteria (Ministry of Education), College of Environmental Science and Engineering, Nankai University, Tianjin, 300071 China; 5 School of Chemistry and Chemical Engineering, Shandong University, Jinan, 250100 China

**Keywords:** Electrocatalysis, Pollution remediation, Electrocatalysis

## Abstract

Due to environmentally friendly operation and on-site productivity, electrocatalytic singlet oxygen (^1^O_2_) production via O_2_ gas is of immense interest in environment purification. However, the side-on configuration of O_2_ on the catalysts surface will lead to the formation of H_2_O, which seriously limits the selectivity and activity of ^1^O_2_ production. Herein, we show a robust N-doped CuO (N–CuO) with Pauling-type (end-on) adsorption of O_2_ at the N–Cu–O_3_ sites for the selective generation of ^1^O_2_ under direct-current electric field. We propose that Pauling-type configuration of O_2_ not only lowers the overall activation energy barrier, but also alters the reaction pathway to form ^1^O_2_ instead of H_2_O, which is the key feature determining selectivity for the dissociation of Cu–O bonds rather than the O–O bonds. The proposed N dopant strategy is applicable to a series of transition metal oxides, providing a universal electrocatalysts design scheme for existing high-performance electrocatalytic ^1^O_2_ production.

## Abstract (web summary)

Side-on configuration of O_2_ on the catalysts conventionally leads to reduction of O_2_ to water. Here, the authors propose a nitrogen doping strategy with Pauling-type adsorption of O_2_ for selective electrocatalytic singlet oxygen production.

## Introduction

Global environmental deterioration has been recognized as one of the most important challenges in the 21st century, especially refractory organic contaminants in environmental water adversely affect the ecological system and potentially threaten human health^1^. Advanced oxidation processes (AOPs) are state-of-the-art water treatment technologies, which can achieve deep oxidation of refractory organic pollutants because of the production of reactive oxygen species (ROS) such as hydroxyl radicals (•OH), hydrogen peroxide (H_2_O_2_), singlet oxygen (^1^O_2_), superoxide radicals (O_2_^•−^) and so on^2,3^. As one of the reactive ROS, ^1^O_2_ has become a favorable candidate for the treatment of refractory organic pollutants in the environmental field because of its mild oxidation ability (2.2 V vs NHE) with aromatic organic compounds, higher tolerance to water matrixes, inhibited generation of halide disinfection byproducts and its relatively long lifetime (over an hour in gas phase and 10^−6^–10^−3^ s in solution)^4–7^. The mild oxidation ability of ^1^O_2_ can selectively oxidize unsaturated organics through electrophilic addition and electron abstraction reaction^8^. Hence, ^1^O_2_ is an effective ROS to remove aromatic organic pollutants from wastewater. Greater recognition of the conspicuous significance of ^1^O_2_ has inspired research for more effective ^1^O_2_ production. At present, the common approaches of ^1^O_2_ production in the practical treatment of wastewater by AOPs mainly include the activation of H_2_O_2_ and peroxymonosulfate^9,10^. However, despite the substantial development of ^1^O_2_ production, the dependence of external chemicals, the introduction of potential emerging pollutants and complicated operation have thus far limited their practical applications. Therefore, the direct chemical synthesis of ^1^O_2_ from the ground state oxygen molecules via photocatalysis and electrocatalysis (EC) could be a potential alternative strategy for ^1^O_2_ production, due to the environmentally friendly operation and on-site productivity^11,12^. In particular, electrocatalytic ^1^O_2_ production, a highly automated, straightforward and propitious technology, can offer a more accessible ^1^O_2_ production pathway and is more promising for industrial scale-up, has obtained extensive research interest. However, various ROS will be involved in EC process due to the low selectivity of O_2_ activation, which seriously limits the application of ^1^O_2_ production^13–15^.

1 $${{{{{{\rm{O}}}}}}}_{2}+\ast \to {}{\ast }{{{{{\rm{O}}}}}}_{2}$$

2 $${\ast }{{{{{\rm{O}}}}}}_{2}+{{{{{{\rm{H}}}}}}}^{+}+{{{{{{\rm{e}}}}}}}^{-}\to {}{\ast }{{{{{\rm{O}}}}}}{{{{{\rm{OH}}}}}}$$

3 $${\ast }{{{{{\rm{O}}}}}}{{{{{\rm{OH}}}}}}\to {}^{\ast }{{{{{\rm{O}}}}}}+{}{\ast }{{{{{\rm{O}}}}}}{{{{{\rm{H}}}}}}$$

4 $${\ast }{{{{{\rm{O}}}}}}{{{{{\rm{H}}}}}}+{{{{{{\rm{H}}}}}}}^{+}+{{{{{{\rm{e}}}}}}}^{-}\to {{{{{{\rm{H}}}}}}}_{2}{{{{{\rm{O}}}}}}+\ast$$

5 $${{{{{{{\rm{O}}}}}}}_{2}}^{\cdot -}/{{{{{{\rm{HO}}}}}}}_{2}\cdot+{{{{{{{\rm{O}}}}}}}_{2}}^{\cdot -}/{{{{{{\rm{HO}}}}}}}_{2}\cdot \,\to {{{{{{\rm{H}}}}}}}_{2}{{{{{{\rm{O}}}}}}}_{2}+{}^{1}{{{{{\rm{O}}}}}}_{2}$$

**Fig. 1:**
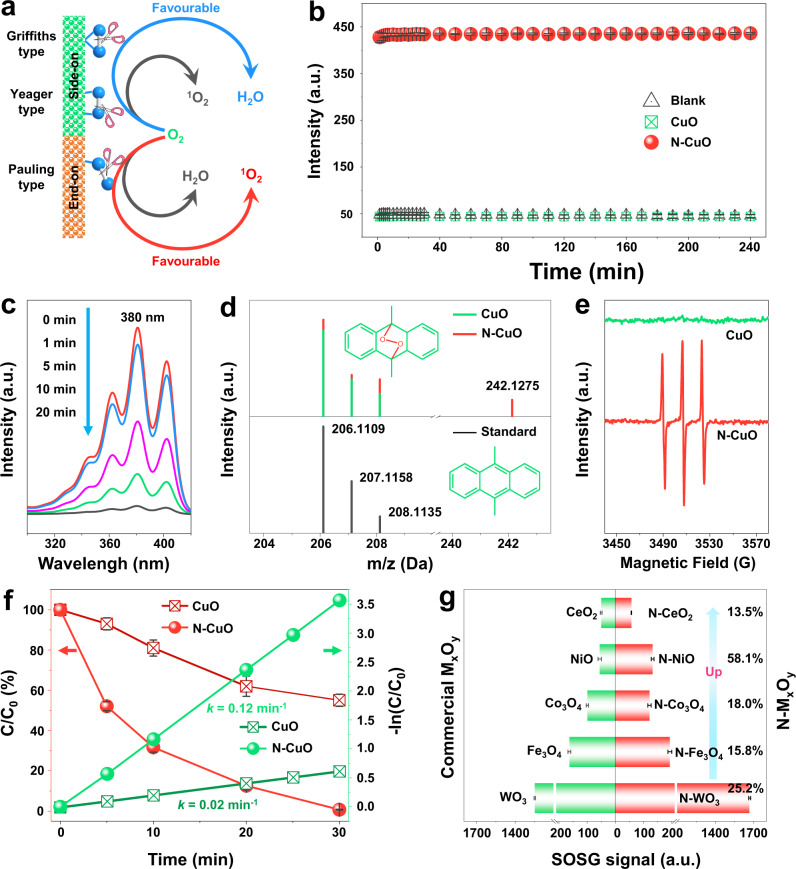
Electrocatalytic performance of N–CuO towards ^1^O_2_ production. **a** Schematic of the molecular oxygen activation pathway of “side-on” and “end-on” adsorption configuration on catalyst surface. **b** Fluorescence response of SOSG upon treatment with CuO and N–CuO within 240 min (λ_ex_ = 504 nm and λ_em_ = 525 nm). **c** Excitation and emission spectra of DMA in 10 wt% acetonitrile in H_2_O upon treatment with N–CuO system. The excitation was recorded from 280 to 420 nm with λ_em_ = 425 nm. **d** HR-MS chromatogram of the typical DMA-O_2_ from the oxidation of DMA in CuO and N–CuO EC system. **e** EPR spectra in CuO and N–CuO EC system using TEMP as trapping agents. **f** Degradation and first-order reaction kinetics of SMX removal in CuO and N–CuO EC system. **g** Comparison of electrocatalytic performances of metal oxides and the corresponding N-doped metal oxides in the ^1^O_2_ production process. Error bars represent the standard deviation from triplicate experiments.

Generally, O_2_ can be adsorbed on the metal oxide or sulfide electrocatalysts surface in “side-on” adsorption configuration (including Griffiths-type and Yeager-type) in the electrocatalytic O_2_ activation process (Fig. 1a)^16^. The “side-on” adsorption configuration can facilitate the cleavage of O–O bonds, leading to the formation of H_2_O (Eqs. (1–4))^17–19^, which are unfavorable for the ^1^O_2_ production. Compared to the cleavage of O–O bonds, “end-on” adsorption (Pauling-type) configuration can minimize O–O bond breaking, leading to the cleavage of M–O bond rather than O–O bond, which will ultimately prevent the cleavage of O–O bonds and facilitate the formation of –O–O– intermediates (the most important intermediates of ^1^O_2_)^20^. Furthermore, the reduction or disproportionate reaction of –O–O– intermediates will generally form the ^1^O_2_ (Eq. (5))^21^. In this case, we can imagine that if O_2_ molecules could be bonded with metal sites by the “end-on” adsorption configuration, this would give rise to a high probability of ^1^O_2_ production, thanks to the reduction or disproportionate reaction of –O–O– intermediates (Eq. (5))^21^. Benefiting from the regulation of the electronic structure by non-metal heteroatom doping engineering, the catalytic activity and selectivity can be improved by regulating binding strength and adsorption configurations of reactants on the surfaces of catalysts. For instance, P-doped CoO-nanoparticles could promote its electrocatalytic activity for oxygen evolution reaction (OER) due to the in-situ formation of active Co–OOH species and the suitable regulation of free energy for adsorption of the OER intermediates^22^. Enlightened by the above analysis, controllable synthesis of local coordination environment of metal center with non-metal heteroatom-doping is highly desirable to improve the ^1^O_2_ generation in EC process.

Herein, we develop an N-doped CuO electrocatalyst for direct electrocatalytic ^1^O_2_ production by using oxygen gas under a direct current (DC) electric field, in which the surface local coordination environment of Cu–O_4_ is regulated to N-Cu-O_3_ moiety. Results elucidats that the selective generation of ^1^O_2_ is achieved in EC process by tailoring the electronic structure and coordination environment of N-doped CuO, which is in sharp contrast to the •OH intermediates generated in the formation of H_2_O in undoped CuO electrocatalytic system. Combining experimental and theoretical investigations, it is found that the adsorption of O_2_ on N–Cu–O active sites is “end-on” type, which promotes the formation of •OOH, leading to an efficient pathway for ^1^O_2_ selective generation. The concept of using non-metal heteroatom doping metal oxides to simultaneously boost ^1^O_2_ selective production shall provide a design guide to develop more advanced electrocatalytic systems for extensive applications.

## Results

### Electrocatalytic ^1^O_2_ production on N-doped CuO

N-doped CuO was prepared by an acetamidine hydrochloride-assisted (C_2_H_6_N_2_·HCl) oxidation etching strategy (Supplementary Fig. 1). The nitrogen-doping level was modulated by tuning the amounts of C_2_H_6_N_2_·HCl from 0 to 8.0 mmol, whereas that of the Cu_2_O precursor and NaOH concentration was maintained at 40.0 mg and 0.13 mol L^−1^, respectively. The optimized N-doped CuO under the C_2_H_6_N_2_·HCl concentration of 0.2 mmol L^−1^ and N content of 2.0 wt% was denoted as N–CuO, and CuO without N doping was prepared as references.

The ^1^O_2_ production performance was evaluated in an aqueous solution (50.0 mmol L^−1^ Na_2_SO_4_, pH ~ 3.0 and applied potential of 3 V) by using multiple approaches, and the performance of N–CuO (Fig. 1b–e) was found to be strikingly different from that of CuO. Singlet oxygen sensor green (SOSG) was employed to monitor ^1^O_2_ generation due to its high selectivity and activity to ^1^O_2_ but not for other ROS^23^. Strikingly, for N–CuO, an obvious and rapid turn-on fluorescence intensity (at 529 nm) of SOSG is observed in the EC process, indicating its excellent ^1^O_2_ generation performance. While for CuO, no obvious change of fluorescence intensity at 529 nm of SOSG is observed, which is very close to the blank experiment (without catalysts), indicating that the N dopant on CuO surface is responsible for the enhanced ^1^O_2_ generation activity and selectivity (Fig. 1b). Moreover, the negligible decay of SOSG fluorescence intensity in N–CuO system is observed for 240 min (Fig. 1b), which confirms the robust stability during long-term operation. Further receivable evidence was displayed based on the determination of indicative products from the specific reactions between ^1^O_2_ and classical chemical probe 9,10-dimethylanthracene (DMA), as they would form the corresponding endoperoxide (DMA-O_2_)^21^. The fluorescence intensity of DMA in the presence of N–CuO decreases rapidly, while the CuO system decreases slowly, indicating the ROS generated in the N–CuO system have higher reaction rate with DMA and might be the ^1^O_2_ (Fig. 1c and Supplementary Fig. 2). The high-resolution mass spectrometry (HR-MS) was carried out to verify the ROS species reacted with DMA in a different system, and the HR-MS peaks of DMA-O_2_ located at 242.1275 Da in N–CuO system is appeared while that of CuO system is negligible, clearly validating the generation of ^1^O_2_ (Fig. 1d).

Furthermore, electron paramagnetic resonance (EPR) measurements were employed to gain more substantive evidence for the ROS species in different systems. Figure 1e presents that the ^1^O_2_ is produced through the EC process using N–CuO catalysts, as confirmed by when the trapping agent is 2,2,6,6-tetramethyl-4-piperidinol (TEMP). Instead, •OH is negligible produced within the N–CuO system, completely demonstrating the superior selectivity of N–CuO catalysts in the ^1^O_2_ production (Supplementary Fig. 3). In addition, the amount of •OH formed in two systems was quantified by the terephthalic acid (TA, which reacts readily with •OH to produce a highly fluorescent product 2-hydroxyterephthalic acid) fluorescence colorimetry (Supplementary Fig. 4), and the •OH formation of CuO system is 170 nmol L^−1^, which is in sharp contrast to the fact that there is almost no •OH in N–CuO system, in view of the fact that the •OH formation in N–CuO system is almost consistent with the blank experiment. The generation of •OH may be due to the Fenton-like reaction in CuO system during 4*e*^−^ oxygen reduction reaction (ORR) process^24,25^.

As ^1^O_2_ is an important ROS for electron-rich organic pollutants degradation, the degradation performance of CuO and N–CuO was investigated with sulfamethoxazole (SMX) as a target pollutant^11^. As shown in Fig. 1f, compared with the inferior SMX removal achieved by CuO, almost total degradation of SMX is achieved within 30 min in the N–CuO system. The degradation kinetic model (*k* value) of the N–CuO system based on the degradation rate is 0.12 min^−1^, and almost 6.0 times higher than that of CuO system. Notably, the optimized N–CuO catalyst with a TOF of 0.41 min^−1^ for SMX degradation exhibits the better catalytic oxidation performance among reported catalysts (Supplementary Table 1). Especially, the N–CuO system achieves the SMX mineralization by means of total organic carbon (TOC) removal rate of 22.3% within 30 min, even its TOC removal rate of SMX reaches 18.0% after 5 cycles (Supplementary Figs. 5 and 6). Strikingly, the unforeseen stability at pH value up to 7.0 greatly expands the use of catalysts in the acidic and neutral environment. And the degradation efficiency of SMX decreases when pH value exceeds 7.0, which might be due to the decline of protons in the solution (Supplementary Fig. 7) (Supplementary Note 1)^26^. In light of the selectivity of ^1^O_2_ for the removal of electron-rich substances, the removal of sulfisoxazole (SFX) and sulfadiazine (SDZ) was investigated. Results demonstrate that the degradation efficiencies of SFX and SDZ are 91.1 and 95.7% after 30 min, and the *k* values are 0.08 min^−1^ and 0.10 min^−1^, showing the efficient removal ability of N–CuO system for electron-rich pollutants (Supplementary Fig. 8). Expectedly, the removal ability of N–CuO system for neutral (ciprofloxacin) and electron-deficient (methylrosanilnium chloride) pollutants is inferior than that for electron-rich pollutants (e.g., SMX, SFX, and SDZ), which is consistent with the previous report on the removal of electron-rich pollutants by ^1^O_2_ (Supplementary Fig. 9)^27^.

Importantly, the N dopant strategy is universal for boosting ^1^O_2_ generation performance in other metal oxides EC system. As shown in Fig. 1g, we studied the ^1^O_2_ generation performance via a variety of commercial metal oxides and the corresponding N dopant catalysts (such as CeO_2_, NiO, Co_3_O_4_, Fe_3_O_4_, and WO_3_) with similar N dopant methods. Results validate that these catalysts have different degrees of enhancement of ^1^O_2_ generation, elucidating that N dopant strategy is universal and feasible to promote the formation of singlet oxygen in EC system with N dopant strategy. Furthermore, the facet effect of N–CuO was investigated, and results show that N-doping effect accounts for the higher catalytic performance of N–CuO than that of facet effect (Supplementary Figs. 10–13).

### Characterization of N–CuO

**Fig. 2:**
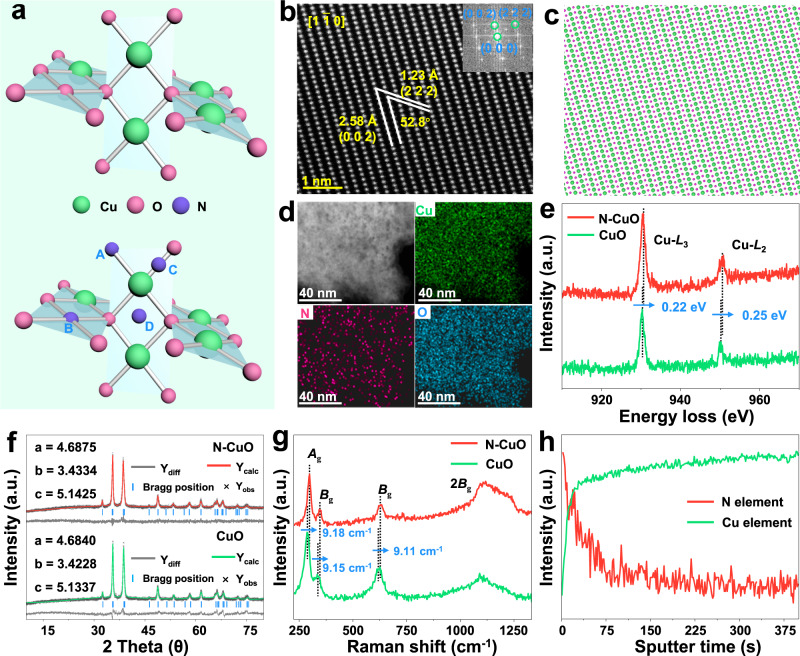
Structural characterization of N–CuO. **a** Schematic presentation of CuO structure (Top: CuO, Bottom: Nitrogen substitution site.). **b** HAADF-TEM image of N–CuO. Inset is the corresponding FFT pattern. The corresponding atomic model (**c**) and the corresponding elemental mapping images (**d**) of N–CuO. **e** Cu *L*-edge EELS spectra of CuO and N–CuO. Refined XRD profile (**f**) and Raman spectra (**g**) of the CuO and N–CuO. **h** TOF-SIMS of N–CuO.

Given the satisfactory ^1^O_2_ production performance of N–CuO, the structural characteristics of the as-synthesized catalysts were further studied. According to the structure of CuO, there are four possible positions (substitutional N in positions A and B, and interstitial N in positions C and D) of N species doped in CuO (Fig. 2a). Unambiguous identification of the N species is rather vital for the design of catalyst and unraveling the reaction mechanism in ^1^O_2_ generation, because the chemical composition, local environment and electronic properties of active sites on catalytic surfaces can regulate their reactivity and selectivity in reaction process.

After etched by acetamidine hydrochloride, the obtained N–CuO nanocrystals become a surface hierarchical cube structure (Supplementary Figs. 14–16). The clear lattice fringes with *d*-spacings of 2.58 and 1.23 Å in the high angle annular dark-field scanning transmission electron microscopy (HAADF-STEM) image indicate the (002) and (222) planes of N–CuO and the corresponding fast Fourier transform (FFT) image shows the high orientation along the [1$\bar{1}$0] projection (Fig. 2b). Though there appears a slight lattice distortion due to the N dopant compared with the crystalline lattices of CuO (Supplementary Fig. 17), the HAADF-TEM image of N–CuO is identical to the regular arrangement of Cu sites in CuO and matches well with the crystal structure of CuO as simulated in Fig. 2c, suggesting nitrogen doping does not cause the structural phase transition^28^. Moreover, the HAADF-TEM reveals a regular periodic arrangement of Cu atoms without obvious vacancy defects in N–CuO, further confirming the structural stability and robustly excluding the possibility of copper substitutions by nitrogen of N–CuO sample. The corresponding elemental mapping and energy dispersive X-ray spectrum (EDX) elemental mapping of HAADF-TEM verify that Cu, O, and N elements are uniformly distributed on the surface of N–CuO (Fig. 2d and Supplementary Fig. 18), indicating the successful incorporating of N^29^. And the N content is ~2.0 wt% measured in the EDX result. We further investigated the N–CuO by electron energy-loss spectroscopy (EELS) in area-scan mode, indicating the presence of N that changed the excitation of the Cu *2p*_1/2_ or Cu *2p*_3/2_ electrons from the inner shell into the first available unoccupied, which might be a result of Cu–N interaction on the surface (Fig. 2e and Supplementary Fig. 19)^30^.

The crystal structure of CuO and N–CuO was further studied by XRD and the refined XRD patterns of N–CuO and CuO, which shows that the phase of CuO after N dopant has not changed, and it is consistent with the powder XRD result (Supplementary Fig. 20 and Fig. 2f)^31^. However, the lattice constant of N–CuO (*a* = 4.6875, *b* = 3.4334 and *c* = 5.1425) are slightly larger than those of pristine CuO (*a* = 4.6840, *b* = 3.4228 and *c* = 5.1337), further suggesting that N dopant leads to lattice distortion. Similarly, the Raman peaks of N–CuO appear blue shift compared with CuO, which is due to the increase in grain size along with the N doping (Fig. 2g)^32^. To further identify the existence form of N species, the time-of-flight secondary ion mass (TOF-SIMS) spectrometry of the N–CuO was employed. Peaks at 78.9–79.04, 79.9, and 80.9 Da are ascribed to CuNH_2_^+^, CuNH_3_^+^, and CuNH_4_^+^ respectively, suggesting that N element is bonded to Cu in the form of enhanced chemical bond, rather than a simple physical or chemical adsorption process (Supplementary Fig. 21). The distribution of N element on the surface and subsurface of N–CuO was analyzed by the ion sputtering mode of TOF-SIMS (Fig. 2h). With the increase of sputtering depth, the relative intensity of nitrogen on CuO decreases, but the signal of N still remained, validating that N atoms are major present only on the surface and subsurface of CuO, which elucidates that N substitution might occur on the surfaces of catalysts. These results strongly suggest that N species enter the CuO lattice in the form of interstitial substitution or oxygen substitution, rather than simply physical adsorption.

**Fig. 3:**
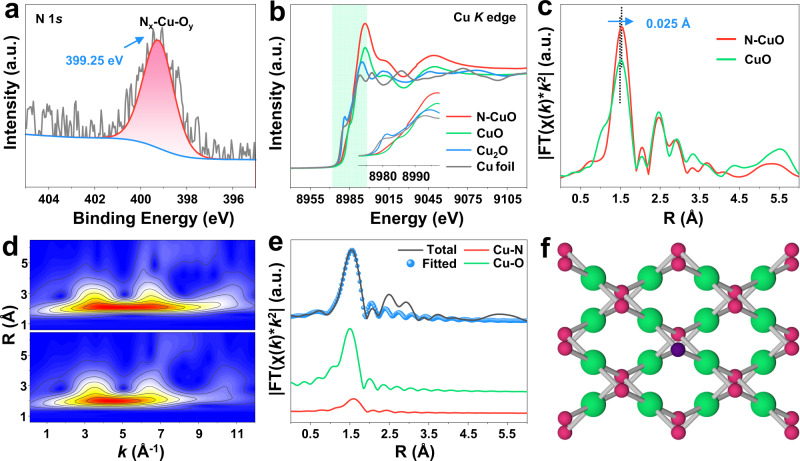
Charge distribution via N-doping strategy. **a** High-resolution N 1*s* XPS spectrum of N–CuO. **b** Normalized Cu *K*-edge XANES spectra of N–CuO and its reference samples (Cu foil, Cu_2_O and CuO). **c, d** Fourier transform-EXAFS spectra (**c**) and WT-EXAFS plots (**d**) from Cu *K*-edge XANES spectra. **e** EXAFS fitting analysis of N–CuO at R space. **f** Schematic optimized atomic coordination model of the N–CuO from EXAFS data.

The chemical and electronic states of N–CuO nanocrystal were further confirmed to understand how the N doping strategy modulates the property of catalysts. The X-ray photoelectron spectroscopy (XPS) analysis of Cu 2*p* and O 1*s* show the peaks for Cu and O elements of CuO (Supplementary Fig. 22), which is consistent with previously reports^33^. The binding energy of the N species is significantly lower than those of NO (402.7 eV), NO_2_ (404.3 eV), and NO_3_^−^ (407.3 eV) and yet is closer to that of NH_3_ (398.7 eV), suggesting that the valence state of the N species may be between −2 and −3 (Fig. 3a). That can completely rule out the positions B and C in Fig. 2a^34^. For the interstitial N (position D in Fig. 2a), however, the density functional theory (DFT) calculations show that it would be unstable (Supplementary Fig. 23). Therefore, the position A (N atoms substitute for the surface O atoms) is the most likely doping site in N–CuO (Fig. 2a).

To further gain insight into the Cu coordination environment in N–CuO, the pre-edge features of X-ray absorption near edge structure (XANES) and extended X-ray absorption fine structure (EXAFS) spectra were collected for Cu elements. The normalized XANES spectra show that the white line intensity of N–CuO is higher than Cu foil and Cu_2_O (Fig. 3b), suggesting that the valence of Cu in N–CuO is about +2 and the insertion of N can change the oxidation state of Cu in our case^35^. The Fourier-transformed (FT) *k*^2^-weighted EXAFS spectra (Fig. 3c) demonstrate that N–CuO only exhibits a prominent peak at 1.54 Å, which is larger than the Cu–O bonding environment of CuO (1.51 Å) and may be attributed to the Cu–N bonds in N–CuO. Moreover, the oscillation curves of *k*-space spectra (Supplementary Fig. 24) confirm the different atomic coordination environment in CuO and N–CuO. In addition, the wavelet transform (WT) contour plots of CuO and N–CuO were further carried out to analyze the localized coordination environments of Cu atoms in both *R* and *k* spaces (Fig. 3d). Evidently, the WT maximum intensity for CuO is 4.5 Å^−1^, while the WT maximum intensity for N–CuO is 5.0 Å^−1^. It is probable that the atomic number difference between O and N leads to the shift of WT maximum^36^.

The quantitative least-squares EXAFS curve-fitting analyses of Cu moieties in N–CuO were further investigated by utilizing two backscattering pathways; that is, Cu–O and Cu–N. The best-fitting analyses obviously display that the main peak at 1.50 Å can be satisfactorily interpreted as Cu–O first-shell coordination (Fig. 3e and Supplementary Fig. 25) and that the shoulder peak at 1.60 Å stems from Cu–N contribution^37^. The coordination numbers for O and N atoms are calculated as 3.0 ± 0.3 and 1.0 ± 0.1 at distances of 1.93 ± 0.01 and 2.03 ± 0.02 Å, respectively (Supplementary Table 2). For comparison, the fitting of CuO results in four oxygen atoms with a distance of 1.97 Å (Supplementary Fig. 25). These results reveal that the Cu atom in CuO coordinates with four O atoms (Cu–O_4_), which is different from the Cu (N–Cu–O_3_) in N–CuO with three O atoms and one N atom (Fig. 3f). Taken together, these spectroscopic findings strongly underpin that the as-obtained N–CuO is composed mainly of Cu–N bonds and Cu-O bonds and the partial surface oxygen atoms in CuO are substituted by N atoms, and the N–Cu–O_3_ may be the active sites in N–CuO for the generation of ^1^O_2_.

### ^1^O_2_ production mechanism of N–CuO

**Fig. 4:**
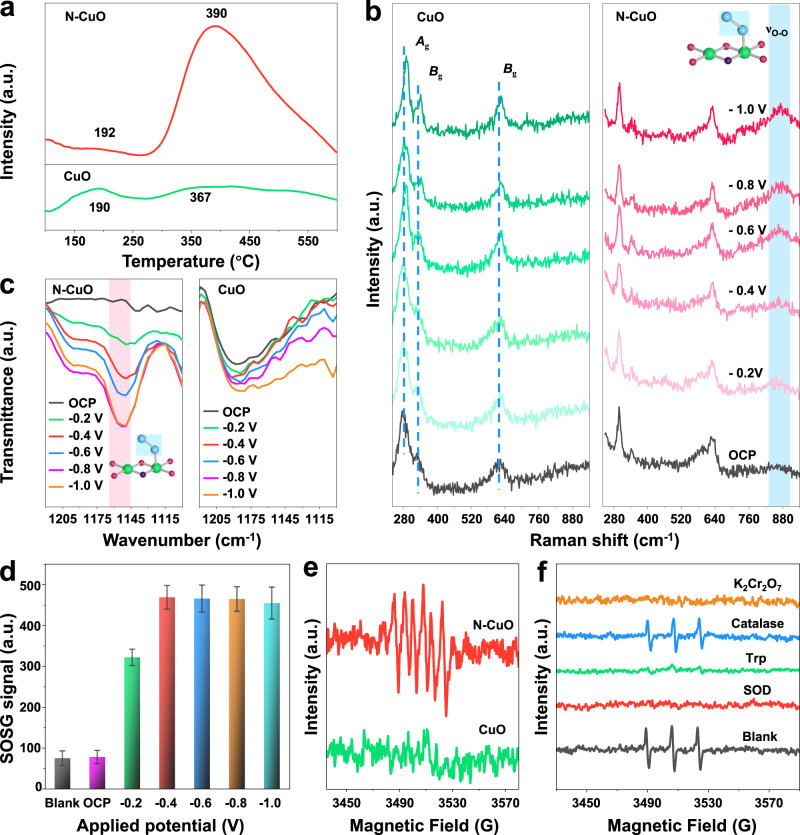
The mechanism for ^1^O_2_ production by the electrocatalytic process from N–CuO. **a** O_2_-TPD profiles of CuO and N–CuO. **b** In situ Raman spectra of CuO and N–CuO in EC system with O_2_-saturated solution. All potentials were referenced to saturated calomel electrode (SCE). **c** In situ FTIR spectra of ^1^O_2_ generation over N–CuO and CuO in EC system with O_2_-saturated solution. **d** Fluorescence response of SOSG under different applied potentials in EC system. Error bars represent the standard deviation from triplicate experiments. **e** EPR spectra for the detection of •OOH/O_2_^•−^ in the presence of DMPO (50 mmol L^−1^). **f** EPR spectra of EC system with different sacrificial agents by using N–CuO.

To evaluate the feasible reaction mechanism and amplify the N-dopant effect for improved ^1^O_2_ generation performance, systematic characterizations were carried out, such as the O_2_ temperature-programmed desorption (O_2_-TPD), the potential-dependent in situ Raman spectroelectrochemistry, in situ Fourier transform infrared (FTIR), EPR spectra and DFT calculations. Firstly, the CuO and N–CuO electrodes were investigated to get further insight into the enhanced electrocatalytic activity after N doping. Results show that N doping results in different electrochemical behavior for the molecular oxygen activation process (Supplementary Figs. 26–28). Then O_2_-TPD spectra were collected to investigate the oxygen species adsorbed on the catalyst surfaces for CuO and N–CuO. As expected, the O_2_-TPD spectrum of N–CuO shows the desorption peak of adsorbed O atoms moves toward higher temperature accompanied with the desorption peak intensity increasing, indicating the superior chemical oxygen adsorption capacity compared with CuO, which demonstrates that N dopant ameliorates the adsorption energy of O_2_ (Fig. 4a)^18,38^. The molecular oxygen activation process is also verified by N_2_- and O_2_-saturation control experiments (Supplementary Figs. 29 and 30). The Raman spectroscopy is based on the vibration of chemical bonds, providing more sensitive and accurate real-time insights into reaction intermediates. Therefore, in situ Raman measurements were conducted to identify the possible intermediates during the ^1^O_2_ generation process. Figure 4b shows the potential-dependent in situ Raman spectroelectrochemistry of CuO and N–CuO in O_2_-saturated 50 mmol L^−1^ Na_2_SO_4_ (pH = 3). The Raman spectrum of the initial sample shows only there are peaks at 295.2, 342.7, and 633.5 cm^–1^ at open circuit potential (OCP), which are attributable to *A*_g_, *B*_g_, and *B*_g_ modes of CuO crystals^39^. The Raman peaks of CuO appear blue shift with the potential decrease to −1.0 V, while no new peaks appear even under more negative potentials. In sharp contrast, as the potential decreases to −0.4 V, the N–CuO sample shows a Raman peak around 872 cm^−1^, which is possibly associated with O–O stretching mode of superoxide species on the surface of N–CuO (Cu^II^–O_2_^•^)^40,41^. Further decreasing the potential, the Raman peak becomes slightly stronger. To exclude the •OOH species adsorbed or desorbed on N–CuO surfaces originating from breakage of water molecules, the control in situ Raman experiment saturated with N_2_ was employed (Supplementary Fig. 31). In this case, the characteristic peak located at 872 cm^−1^ of Cu^II^–O_2_^•^ in the Raman spectrum is not observed, providing strong evidence that the origin of Cu^II^–O_2_^•^ is related to the activation of oxygen molecules.

To further confirm the oxygen adsorption configuration, in situ FTIR experiments were carried out. Similar results explain that there are different O_2_ adsorption configurations on CuO and N–CuO surfaces. The accumulated intermediate species adsorbed on the surface of N–CuO are observed by in situ FTIR. From Fig. 4c, the ^1^O_2_ generation process can be divided into two stages in N–CuO system, namely, the accumulation of intermediate (Cu^II^–O_2_^•^) and the •OOH desorption process. When the potential decreases from open circuit potential (OCP) to –0.8 V, the intensity of peak located at around 1135–1160 cm^−1^ appears and increases, which can be assigned to the stretching mode of –O–O– adsorbed on N–CuO (correspond to the end-on Cu–O_2_^•^ on the surface of N–CuO)^42,43^. That is the accumulation of the intermediate species (Cu^II^–O_2_^•^). While with the further decrease of potential, the intensity of peak barely changes, corresponds to the •OOH desorption process^41,42^. In comparation, no new peaks appear around 1135–1160 cm^−1^ for CuO even at more negative potentials, verifying that there are different O_2_ adsorption configurations on CuO and N–CuO surfaces (Fig. 4c). The ^1^O_2_ semi-quantitative experiment under the corresponding applied potential was carried out, and the fluorescence intensity of SOSG signal corresponds to the Raman peak intensity located at 872 cm^−1^ or FTIR peak located at around 1135–1160 cm^−1^, indicating that the formation of Cu^II^–O_2_^•^ is an important step in the ^1^O_2_ generation process (Fig. 4d).

Considering that •OOH species are the prominent intermediates in the Haber–Weiss cycle, especially in the generation process of ^1^O_2_, we assume that Cu^II^–O_2_^•^ has the step of desorption^10,21^. To estimate this assumption, the EPR spectra were tested and shown in Fig. 4e. DMPO-OOH (α_N_ = 12.9 G, α_H_ = 10.3 G, *g* = 2.0057, a spin adduct derived from DMPO-OOH) signal is detected for N–CuO system, while there is no DMPO-OOH signal in CuO system, confirming that Cu^II^–O_2_^•^ detected in in situ Raman and FTIR spectra may undergo the desorption process. Furthermore, the amount of •OOH produced in this period are then quantified using nitroblue tetrazolium chloride (NBT)^44^. As shown in Supplementary Fig. 32, the results show that at least ~10 μmol L^−1^ •OOH are accumulated in N–CuO system, giving solid evidence for the desorption step of Cu^II^–O_2_^•^ and then the formation of free •OOH. In view of the •OOH is the major intermediate in 2*e*^−^ ORR reaction, we then monitored linear sweep voltammetry (LSV) and ROS generation with the addition of H_2_O_2_ in EC system, confirming that N–CuO could suppress the generation of •OH in N–CuO system and the main source of ^1^O_2_ comes from •OOH (Supplementary Figs. 33–35)^45^. To further clarify the main intermediate accounting for the formation ^1^O_2_ generation, the respective effect of superoxide dismutase (SOD, the scavenger for •OOH), tryptophan (Trp, the scavenger for ^1^O_2_), and catalase (the scavenger for H_2_O_2_) on the EPR signal were investigated. As it is seen in Fig. 4f, catalase shows slight suppression on the signal intensity, while SOD and Trp as scavengers strongly diminish the observed peaks of the 2,2,6,6-tetramethyl-4- piperidinol-N-oxyl radical (TMPN) adduct, displaying the •OOH is the most intermediate for the ^1^O_2_ generation^46^. To further confirm the role of •OOH for the ^1^O_2_ generation, the quenching process of •OOH in the electrocatalysis system was investigated (Fig. 4f)^47^. As potassium dichromate (K_2_Cr_2_O_7_) is able to remove the electron of •OOH, it is determined to be used to investigate the role of •OOH for ^1^O_2_ generation in the N–CuO system^47,48^. The ^1^O_2_ generation had an obvious decrease in the N–CuO system after adding of 5 mmol L^−1^ K_2_Cr_2_O_7_ (Fig. 4f). These results suggest that •OOH is an important intermediate for ^1^O_2_ generation. The results are in concurred with the sacrificial agent experiment in the SMX degradation process, indicating the major ROS involving the ^1^O_2_ and •OOH in EC system (Supplementary Fig. 36). In addition, the contribution of anode is excluded by control experiment (Supplementary Fig. 37). Based on the above analysis in Fig. 4, it is speculated that the most likely ^1^O_2_ generation path could be as follows (Eqs. (6–8))^21,49^:

6 $$\ast+{{{{{{\rm{O}}}}}}}_{2}+{{{{{{\rm{e}}}}}}}^{-}\to {}{\ast }{{{{{{{\rm{O}}}}}}}_{2}}^{-}$$

7 $${\ast }{{{{{{{\rm{O}}}}}}}_{2}}^{-}+{{{{{{\rm{H}}}}}}}^{+}\to \ast+^{\cdot} {{{{{\rm{OOH}}}}}}$$

8 $${{{{{\rm{HOO}}}}}}^{\cdot}+{}{\ast }{{{{{{{\rm{O}}}}}}}_{2}}^{-}\to {{{{{{{\rm{HO}}}}}}}_{4}}^{-}\to {{{{{{\rm{HOO}}}}}}}^{-}+{}^{1}{{{{{\rm{O}}}}}}_{2}$$

**Fig. 5:**
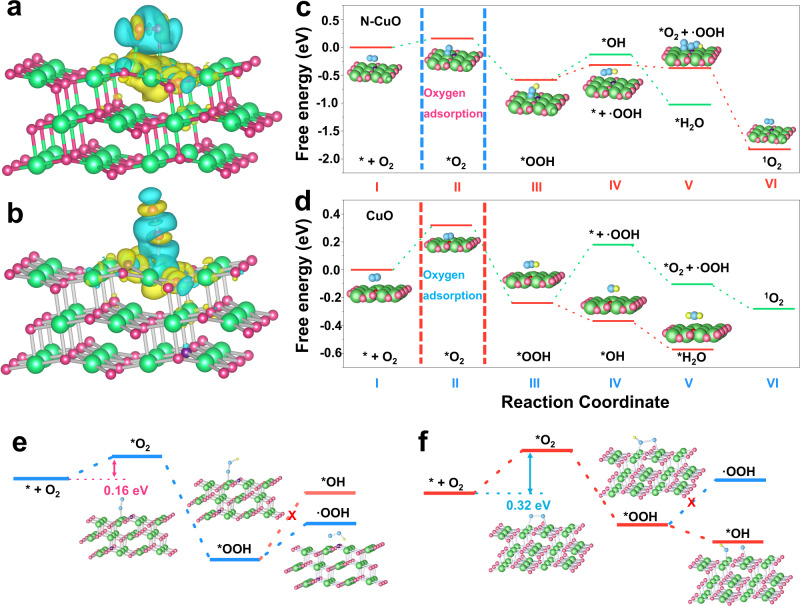
Gibbs free energy calculations. **a**, **b** O_2_ adsorption configurations and calculated differential charge densities on CuO (222) (**a**) and N–CuO (222) (**b**). Free energy diagrams of molecular oxygen activation process for N–CuO (**c**) and CuO (**d**). Key steps of ^1^O_2_ production for **e** N–CuO (222) and **f** CuO (222).

To better understand the effect of O_2_ adsorption configuration on ^1^O_2_ generation at the atomic level, DFT calculations were conducted to study the active sites and the reaction mechanism (Fig. 5). According to the experimental results, the O_2_ adsorption configuration determines the selectivity of the ^1^O_2_ generation. Therefore, we have compared the adsorption energies of different O_2_ adsorption configurations on the CuO and N–CuO surfaces (Supplementary Fig. 38). Results show that the N–Cu–O_3_ sites on the surface of N–CuO are preferential for the Pauling-type adsorption configuration of O_2_, while CuO is more favorable to the O_2_ Yeager-type adsorption on two adjacent Cu atoms around the O coordination. This can be ascribed to the fact that N dopant results in a redistribution of electrons of N–Cu–O_3_ sites on the N–CuO surface, leading to the charge deficiency on Cu atoms (0.84 eV) around the N coordination (Supplementary Figs. 39 and 40) (Supplementary Note 2)^50,51^. The *O_2_ also tend to bond more easily with the N–Cu–O_3_ sites (N–CuO) than the Cu–O_4_ (CuO) sites on account of the change of Cu coordination environment, which helps to lower the *OOH intermediates formation energy in N–CuO; hence, these charge-deficiency Cu atoms (N–Cu–O_3_) in the N–CuO could serve as preferential active sites, which could more effectively absorb the O_2_ via Pauling-type adsorption compared to the Yeager-type adsorption on dual Cu sites with the uniform distribution of localized charge of CuO, hence promoting the formation of *OOH (Fig. 5a). The charge-density difference diagram further unveils that N dopant can result in significant charge transfer from Cu atoms with N coordination to the N and O atoms and affirms the strong interaction between O_2_ in Pauling-type adsorption (Fig. 5a, b and Supplementary Fig. 41) (Supplementary Note 3). These results indicate that the N doping can facilitate the O_2_ Pauling-type adsorption, which might be conducive to the ^1^O_2_ generation.

The Gibbs free energy calculations on the deduced reaction selectivity over the N–CuO and CuO were carried out to gain further insight between the active sites and O_2_ adsorption configuration from a thermodynamic viewpoint. It can be seen that the free energy for absorbed *O_2_ $({\Delta {{{{{\rm{G}}}}}}}_{{\ast {{{{{\rm{O}}}}}}}_{2}})$ is the key bifurcating point to determine whether the ^1^O_2_ generation (Fig. 5c, d). For the N–CuO, the reaction step from absorbed *O_2_ (Pauling-type adsorption) to ^1^O_2_ generation is downhill in the free energy, which is much more favorable thermodynamics for ^1^O_2_ generation (Fig. 5c). The pathway of the *OOH intermediates desorption on the N–CuO is theoretically feasible. In the next step of ^1^O_2_ production, the reaction involving ·OOH and *O_2_ (the reduction or disproportionate reaction, Eq. (8)) is thermodynamically favorable, suggesting that *OOH is the most major intermediate in electrocatalytic production of ^1^O_2_^49^. Remarkably, the ${\Delta {{{{{\rm{G}}}}}}}_{{\ast {{{{{\rm{O}}}}}}}_{2}}$ (Yeager-type adsorption) of the CuO leads to much uphill reaction for molecular oxygen activation. Compared to N–CuO, CuO prones to the dissociation of O–O band. And the dissociation process of *OOH is exoergic, leading to the formation of H_2_O, which is expected to be a favorable step (Fig. 5d).

The Pauling-type adsorption of O_2_ would be more favorable for ^1^O_2_ production because of lower energy barriers (0.16 eV), in comparation with the Yeager-type adsorption mode of CuO (0.32 eV) (Fig. 5e, f). In brief, for the N–CuO, it is theoretically possible that N coordinated Cu atoms with charge deletion could act as active sites to more efficiently stabilize the rate-limiting *OOH intermediates than the CuO. This would help to change the electrocatalytic selectivity and activity of the N–CuO. In addition, though the *OOH desorption process is endothermic on the N–CuO surfaces, it can be thermodynamically feasible at higher potential, while the dissociation of *OOH is thermodynamically favorable for CuO^21^. N dopant alters the exoergic dissociation step to the desorption step by changing the adsorption configuration and hence allowed *OOH desorption to proceed spontaneously thus changing the reaction pathways to form ^1^O_2_ instead of H_2_O in the N–CuO. The stronger O–O bonds on the N–CuO surface lead to easier weakening as well as breakage of the Cu–O bonds in the *OOH desorption step, thus finally decreasing the desorption energy of the *OOH. All these advantages could enable the N–CuO to reach high product selectivity along with high activity for ^1^O_2_ generation.

## Discussion

In summary, the selectivity of the catalyst is related to the adsorbed intermediates and the reaction pathways, then an effective N dopant strategy for improving ^1^O_2_ generation selectivity and activity of metal oxides is demonstrated. The combined characterization results reveal that N species appear in the form of O substitutional N in N–CuO, and the coordination environment of Cu atoms is coordinated with one N and three O, which is identified unambiguously to be the active sites for the O_2_ Pauling-type adsorption and then ^1^O_2_ generation. Furthermore, DFT calculations and spectroscopy characterizations suggest that N dopant results in charge deletion on the Cu atoms surrounding with N atoms, favouring the breakage of the Cu–O bonds rather than O–O bonds, which promotes the formation of *OOH, an important precursor of ^1^O_2_. This approach is general and applicable to a variety of electrocatalysts, including CuO, CeO_2_, NiO, and so on. As an outcome, a 6.0 times faster SMX degradation kinetics in N–CuO than CuO system is obtained. This work represents the collaborative effect between atomic coordination environment and the adsorption configuration of the reactants, which opens up a strategy for designing various nitrogen-doped metal oxides for the electrocatalytic ^1^O_2_ generation reactions and is promising for the application of environmental remediation.

## Methods

### Preparation of the Cu_2_O cubes

Cu_2_O was synthesized by adapted from known literature procedures^52^. In a typical procedure for Cu_2_O, 10.0 mL of 0.1 mol L^−1^ EDTA solution was added to 10.0 mL of 0.1 mol L^−1^ CuSO_4_ solution. The blue color of the CuSO_4_ solution became deep. Then, 10.0 mL of 0.1 mol L^−1^ glucose solution was added to this solution, and the solution was then stirred well for 15 min at room temperature. Finally, 10.0 mL of 1.0 mol L^−1^ NaOH was added to it, and the solution was heated in an oil bath at 80.0 °C for 30 min. The color of the resulting solution changed from blue to green to red. The red particles were collected by centrifugation and washed several times with deionized water and absolute ethanol, and dried well at 60.0 °C overnight for further characterization.

### Preparation of N–CuO

The as-prepared Cu_2_O cubes (40.0 mg) were dispersed into 30.0 mL deionized water containing 4.0 mL NaOH (1.0 mol L^−1^). Then, acetamidine hydrochloride aqueous solution (1.0 mol L^−1^) was added to the above mixture under stirring. After 90 min, the precipitate was collected by centrifugation, washed with water and ethanol several times. The preparation of the working electrode was similar with above method, except immersing the carbon felt (CF, 3 cm × 2 cm) in the above mixture.

### Materials characterization

The morphology and structure of the samples were examined by field emission scanning electron microscope (FESEM; SU-8010), transmission electron microscope (TEM, FEI, USA) and HAADF-STEM energy-dispersive X-ray spectrometry (dual aberration correctors and four energy-dispersive X-ray spectroscopy (EDS) detectors. The EELS spectra under area-scan mode were recorded with an FEI Titan Themis scanning transmission electron microscope operating at 300 kV quipped with a Gatan image filter (GIF) spectrometer. The crystal phases of the samples were analyzed by X-ray diffraction (XRD) by using Rigaku Corporation (Japan) with Cu Kα radiation (λ = 1.54178 Å), operated at an accelerated voltage of 40 kV and an emission current of 40 mA. Raman spectrum was collected on a Renishaw Invia Reflex Raman microscope equipped with a 532 nm excitation laser under the power of 20 mW. Time-of-flight secondary ion mass spectrometry (TOF-SIMS) measurements were carried out using TOF.SIMS.5-NSC instrument (TOF-SIMS 5 iontof, PHI NanoTOFII) allowed the surface chemistry characterization of investigated samples. Surface chemical compositions of catalysts were determined by X-ray photoelectron microscopy (XPS, Thermo Fisher Scientific ESCALAB-250Xi, USA). The binding energies were corrected to C1*s* at 284.8 eV. Electron paramagnetic resonance (EPR, Bruker A300-10/12, Germany) tests were used to characterize ROS in heterogeneous EC process. Total organic carbon (TOC) was analyzed on a Shimadzu VCSH TOC analyzer (Japan).

### Potential-dependent in situ Raman spectroelectrochemistry

Raman spectroscopy was recorded on the same Renishaw InVia reflex Raman microscope under an excitation of 532 nm laser under controlled potentials by an electrochemical workstation (CHI 760E, China). The electrolytic cell was homemade by Teflon. The working electrode was set to keep the plane of the sample perpendicular to the incident laser. Pt wire as the counter electrode was rolled to a circle around the working electrode. Saturated calomel electrode (SCE) was used as the reference electrode.

### Catalytic activity measurements

The evaluation of pollutants degradation performance was performed in a single-cell for electrolyzation in two-electrode system with the cell voltage of 3.0 V^53^. The prepared CF loaded with catalysts was served as cathode equipped with anode using the platinum electrode (2 cm × 1 cm), with a spacing of 1.0 cm between them. Then 65 mL simulated wastewater containing 50.0 mg L^−1^ pollutant (e.g., SMX, SFX, or SDZ) and 50.0 mmol L^−1^ Na_2_SO_4_ solution was electrolyzed under aeration of O_2_ with a flow rate of 100 mL min^−1^. In addition, the pH value of simulated wastewater was adopted to 3 by 0.5 mol L^−1^ H_2_SO_4_ and 0.5 mol L^−1^ NaOH solution.

### Radical quenching tests

Radical quenching tests were conducted to identify the dominant radicals in the EC system with catalase, superoxide dismutase (SOD) and tryptophan (Trp) (radical scavenger for H_2_O_2_, O_2_^•−^ and ^1^O_2_, respectively), which were added before the EC reaction. The radical species were further detected by EPR technology, where 5, 5-dimethyl-1-pyrroline (DMPO) and 2,2,6,6-tetramethyl-4-piperidinol (TEMP) were used as a spin-trapping reagent. The detailed parameters are as follows: 100.0 μL of aqueous suspension of samples were mixed with 1.0 mL of TEMP (50.0 mmol L^−1^) solution. The •OH trapping-EPR test was also performed as described above, except the use of DMPO (50.0 mmol L^−1^) as the spin-trapping agent. As for O_2_^•−^, 1 mL methanol and water (20:1, *v*:*v*) containing DMPO (50.0 mmol L^−1^) were mixed with reaction liquid.

### X-ray absorption fine structure

The Cu *K*-edge X-ray absorption find structure spectra of N–CuO and its references were obtained at 1W1B station in Beijing Synchrotron Radiation Facility (BSRF) under ambient conditions using a transmission mode. The storage ring conditions of BSRF was operated at the energy of 2.5 GeV and a maximum current of 250 mA with double-crystal Si (111) as monochromator for energy selection.

### Computational details

We employed the first-principles^54^ to perform all spin-polarization DFT calculations within the generalized gradient approximation (GGA) using the Perdew–Burke–Ernzerhof (PBE) formulation^55^. We chose the projected augmented wave (PAW) potentials to describe the ionic cores and take valence electrons into account using a plane wave basis set with a kinetic energy cutoff of 400 eV^56,57^. Partial occupancies of the Kohn-Sham orbitals were allowed using the Gaussian smearing method and a width of 0.05 eV. The electronic energy was considered self-consistent when the energy change was smaller than 10^−4^ eV. A geometry optimization was considered convergent when the energy change was smaller than 0.05 eV Å^−1^. Finally, the adsorption energies (*E*_ads_) were calculated as:

9 $${E}_{{{{{{\rm{ads}}}}}}}={E}_{{{{{{\rm{ad}}}}}}/{{{{{\rm{sub}}}}}}}-{E}_{{{{{{\rm{ad}}}}}}}-{E}_{{{{{{\rm{sub}}}}}}}$$

where *E*_ad/sub_, *E*_ad_, and *E*_sub_ are the total energies of the optimized adsorbate/substrate system, the adsorbate in the gas phase, and the clean substrate, respectively. The Brillouin zone integral uses the surfaces structures of 2 × 2 × 1 monkhorst pack K point sampling for structures and we fixed the bottom layers. The free energy (Δ*G*) for elemental reaction step were calculated as:

10 $$\Delta G=\Delta E+\Delta {E}_{{{{{{\rm{ZPE}}}}}}}-T\Delta S$$

where Δ*E* is the difference between the total energy, Δ*E*_ZPE_ and Δ*S* are the differences in the zero-point energy and the change of entropy, *T* is the temperature (*T* = 300 K in this work), respectively.

## Supplementary information

Supporting Information

## Data Availability

The data that support the findings of this study are available from the corresponding author upon reasonable request.
