# Supplementary material for: Pauling-type adsorption of O2 induced electrocatalytic singlet oxygen production on N–CuO for organic pollutants degradation
Source: Nat Commun. 2022 Sep 22;13:5560. doi: 10.1038/s41467-022-33149-4 (PMC9500010; doi:10.1038/s41467-022-33149-4)
Supplement: Supplementary file 1 — Supporting Information [file 41467_2022_33149_MOESM1_ESM.pdf]

Pauling-type adsorption of O_2 induced electrocatalytic singlet oxygen production on N-CuO for organic pollutants degradation

Liangbo Xie[1,6], Pengfei Wang[2,6], Yi Li[1,3], Dongpeng Zhang[4], Denghui Shang[1], Wenwen Zheng[1], Yuguo Xia[5], Sihui Zhan[4*], Wenping Hu[1,3]

[1]Tianjin Key Laboratory of Molecular Optoelectronic Sciences, Department of Chemistry, School of Science, Tianjin University & Collaborative Innovation Center of Chemical Science and Engineering (Tianjin), Tianjin 300072, China

[2]Tianjin Key Lab Clean Energy & Pollutant Control, School of Energy and Environmental Engineering, Hebei University of Technology, Tianjin 300130, China

[3]Joint School of National University of Singapore and Tianjin University, Fuzhou International Campus, Tianjin University, Binhai New City, Fuzhou 350207, China

[4]Key Laboratory of Pollution Processes and Environmental Criteria (Ministry of Education), College of Environmental Science and Engineering, Nankai University, Tianjin 300071, China

[5]School of Chemistry and Chemical Engineering, Shandong University, Shandong 250100, China

[6]These authors contributed equally: Liangbo Xie, Pengfei Wang.

Contributions

S.H. Zhan and Y. Li conceived and designed the research. L.B. Xie carried out the experiment. P.F. Wang and L.B. Xie wrote the manuscript. and D.P. Zhang performed the X-ray absorption analysis. All authors discussed and analysed the data.

Corresponding author

Correspondence to: Sihui Zhan (sihuizhan@nankai.edu.cn)

Contents:

Supplementary Materials and Methods

Supplementary Figures 1 to 41

Supplementary Tables 1 to 2

Supplementary Notes 1 to 3

Supplementary References

Supplementary Materials and Methods

Materials and chemicals

Sulfamethoxazole (SMX, *M*w = 253.28 Da, ≥98%), methylrosanilnium chloride (MC, ≥90%), , hydrogen peroxide (30% (w/w) in H_2O), Cerium (IV) sulfate hydrate ($Ce(SO_4)_2 \cdot H_2O$, 99.99%), copper oxide (CuO), acetamidine hydrochloride-assisted ($C_2H_6N_2 \cdot HCl$), catalase, 2,2,6,6-tetramethyl-4-piperidinol (TEMP, ≥99%), 5, 5-dimethyl-1-pyrroline (DMPO), superoxide dismutase (SOD) and tryptophan (Trp, ≥99.5%) were purchased from Aladdin Biochemical Technology Co., Ltd. Glucose, copper sulfate pentahydrate ($CuSO_4 \cdot 5H_2O$) and ethylenediamine tetraacetic acid (EDTA) were obtained in Tianjin Jiangtian Chemical Technology Co. Ltd. Sulfamethoxazole (SMX), sulfisoxazole (SFX), sulfadiazine (SDZ), nitroblue tetrazolium (NBT) and sodium sulfate (Na_2SO_4) were purchased from Beijing J&K Scientific Ltd. 9,10-dimethylanthracene (DMA), terephthalic acid (TA) and 2-hydroxyterephthalic acid Singlet Oxygen Sensor Green (SOSG) were purchased from meilunbio®. Ultrapure water (18.2 MΩ·cm) was used throughout the work. The carbon felt (CF) was supplied by Shanghai Qijie Carbon Material Co. Ltd.

Experimental Procedures

OCT-Cu_2O: The synthesis of OCT-Cu_2O nanocrystals was performed according to our previous work[1]. In detail, 2.0 mmol $Cu(NO_3)_2$ was dissolved with 20 mL water in a glass vial and put in a water bath at 55 °C, then 0.7 g PVP was subsequently added. When PVP thoroughly dissolved in the solution, 6 mL of 1 mol·L^{-1} NaOH aqueous solution was added under vigorous stirring for 10 min. Then, 2 mL of 0.6 mol·L^{-1} ascorbic acid aqueous solution was introduced as a reductant and kept the reaction for 30 min. After the reaction, the products were centrifuged and washed with water and ethanol for several times, then dried and saved under vacuum at ambient temperature for further use.

DOD-Cu_2O: The RD-Cu_2O nanocrystals were synthesized via a revised protocol using glucose as reductant[1]. In detail, 1 mmol $CuSO_4 \cdot 5H_2O$ was dissolved with 15 mL of water in a glass vial and put in a water bath at 80 °C, then a mixed solution contained 2 mL of oleic acid and 5 mL of ethanol was added under magnetic stirring for 30 min. After that, 5 mL of 1 mol·L^{-1} NaOH aqueous solution was subsequently added and maintained for 10 min. Then kept the reaction for 3 h at 80 °C. The color of suspension gradually turned from deep blue to brick red. The brick red products were collected by centrifugation and carefully washed with cyclohexane and ethanol for dozens of times to remove residual oleic acid, and finally dried and saved under vacuum at ambient temperature for further use.

CuO-140: In the synthesis, 0.125 g $CuSO_4 \cdot 5H_2O$ was dissolved into deionized water (5 mL) to form a clear solution and aged for 10 min, and then 5 mL NaOH solution (0.4 mol·L^{-1}) was added into the $CuSO_4$ solution[2]. Subsequently, the mixture was stirred for 20 min, transferred into a Teflon-lined stainless-steel autoclave (20 mL), and treated hydrothermally at 140 °C for

10 h. After the autoclave was cooled to room temperature, the precipitate was collected by centrifugation, washed with deionized water and anhydrous ethanol, and then dried in a vacuum oven at 50 °C for 8 h.

CuO-200: In the typical procedure, $Cu(NO)_3\cdot3H_2O$ (6.04 g) was dissolved in deionized water (50 mL)[3]. Aqueous NaOH solution (5 mol·L^{-1}, 50 mL) was then added to the above solution under vigorous stirring. The reaction system was transferred into a Teflon-lined stainless-steel autoclave and heated to 200 °C, which was maintained at this temperature for 2 h. The product was collected and washed with deionized water and ethanol, three times each, and then dried in a vacuum oven at 50 °C for 8 h.

N-CuO-x: The as-prepared Cu_2O (40.0 mg) or CuO (22.2 mg) was dispersed into 30.0 mL deionized water containing 4.0 mL NaOH (1.0 mol·L^{-1}). Then, acetamidine hydrochloride aqueous solution (1.0 mol·L^{-1}) was added to the above mixture under stirring. After 90 min, the precipitate was collected by centrifugation, washed with water and ethanol several times.

ROS Quantitative analysis. Nitroblue tetrazolium (NBT) (2.5×10^{-2} mmol·L^{-1}, exhibiting an absorption maximum at 259 nm) was used to determine the amount of $O_2^{\bullet-}$ generating from CuO and N-CuO EC system. The production of $O_2^{\bullet-}$ was quantitatively analyzed by detecting the concentration of NBT with UV-vis spectrophotometry[4-6]. TA (0.5 mmol·L^{-1} in a 2 mmol·L^{-1} NaOH solution) reacted readily with •OH to produce a highly fluorescent product, 2-hydroxyterephthalic acid, which was employed as a probe molecule to determine the amount of •OH generating from CuO and N-CuO EC system[7,8]. The production of •OH was quantitatively analyzed by detecting the concentration of 2-hydroxyterephthalic acid (fluorescence peak at about 425 nm by excitation with the wavelength of 315 nm) with FLS980

fluorescence spectrometer (Edinburgh Instruments Ltd.). The quencher group for $^1O_2$ was typically a substituted anthracene derivative 9,10-dimethylanthracene (DMA), which formed the corresponding endoperoxide[9]. DMA itself was used directly as an $^1O_2$ probe based on the decrease of absorbance as a sign for the presence of $^1O_2$. We added 1 mL DMA (3.2 μmol·L^{-1}) to 1.0 mL reaction liquid. DMA was excited at 380 nm and the emission was detected at 425 nm.

The concentration of H_2O_2 produced in heterogeneous EC process was quantified by a traditional cerium sulfate titration method[10]. Briefly, the H_2O_2 concentration of measurement was performed according to the mechanism of coloration reaction of Ce^{4+}(yellow)/Ce^{3+}(colorless) as shown in Eq. S1.

$$2Ce^{4+} + H_2O_2 \rightarrow 2Ce^{3+} + 2H^+ + O_2 \quad (1)$$

The measurement method was as follows: 500 μL different concentrations of H_2O_2 (0.1 – 1.2 mmol·L^{-1}) were added to 2.0 mL yellow transparent $Ce(SO_4)_2$ solution (0.1 mmol·L^{-1}), respectively. The $Ce(SO_4)_2$ solution was obtained by dissolving 0.01 mmol $Ce(SO_4)_2$ in 100.0 mL 0.5 mol·L^{-1} sulfuric acid solution. The absorbance of above mixed solution was determined by UV-vis spectrometry (the wavelength was at 316 nm).

SMX measurement

SMX concentration was detected by Agilent HPLC (Agilent 1100)[11]. Sample of 50 μL was injected for each measurement at a temperature of 20 °C. Separation was conducted in an XDB-C18 column (5.0 μm, 4.6 × 150 mm, Agilent) with a mobile phase of 40% acetonitrile and 60% phosphoric acid (*v*/*v*) at a flow rate of 2 $mL \cdot min^{-1}$. SMX was quantified at adsorption wavelength of 277 nm and detected at a retention time of 1.83 min.

Liquid chromatography-mass spectrometry detection. A quadrupole time-of-flight liquid chromatography-mass spectrometry (QTOF LC-MS) system (Agilent 6545 series) was used to identify the oxidation products of SMX. The HPLC system was equipped with EclipsePlus C18 column (2.1 × 50 mm, 1.8 μm particle size) for separation. The mobile phase for the separation of SMX consists of Milli-Q water (A) and acetonitrile (C), which varied linearly from 20/80 to 95/5 (*v*/*v*, A/C) from 0 min to 10 min; the flow rate was set as 0.2 $mL \cdot min^{-1}$. In following MS analysis, ions were generated by an electrospray ion source (ESI). The MS analysis parameters for SMX were set as follows: positive mode (ESI^+); gas temperature, 320 °C; drying gas, 8 L min^{-1}; capillary, 3000 V; nebulizer, 35 psi; skimmer voltage, 65 V; fragmentor 175 V. Oxidation products of SMX were analyzed by LCMS/MS.

DMA measurement

The MS signal of 9,10-dimethylanthracene (DMA) and 9,10-dimethylanthracene-endoperoxide (DMA-O_2) were recorded in a mass range of 100 – 450 m/z using the APCI in the positive ion mode. The MS signal was used to identify the retention times for DMA and DMA-O_2. The extent of the transformation of DMA to DMA-O_2 was determined from the absorbance at 258 nm and the molar absorption cofficients $\epsilon_{\mathrm{DMA,\,258\,nm}}$ and $\epsilon_{\mathrm{DMA\text{-}O_2,\,258\,nm}}$.

***In situ* FTIR spectroscopy.** *In situ* FTIR spectra were obtained on a Nicolet 6700 FTIR spectrometer with silicon as the prismatic window. A thin layer of gold film was chemically deposited on the surface of the silicon prismatic before each experiment. Then, 20 μL sample ink was carefully dropped on the surface of the gold film, together serving as the working electrode. The Pt sheet and saturated calomel electrode (SCE) were used as the counter electrode and the reference electrode, respectively.

Magnetic properties measurements. Magnetic properties were studied with a vibrating sample magnetometer (VSM, VersaLabTM 3T, Cryogenfree Vibrating Sample Magnetometer). For zero field cooled (ZFC) and field cooled (FC) measurements, vacuum dried samples were demagnetised at 293 K by setting an initial field of 300 kOe and decreasing the field stepwise to zero by oscillating at 200 $Oe\cdot s^{-1}$. Samples were cooled down to 2 K via a cryocooler based cooling system at zero field. Then an external field of 300 Oe was applied and the samples were heated to 300 K at 1 $K\cdot min^{-1}$ and again cooled down to 2 K at 1 $K\cdot min^{-1}$ in the 300 Oe field. The magnetization was measured by vibrating the samples at 40 Hz. One data point was delivered for M measured within 1 s (averaging time).

Electrochemical measurements. All the electrochemical tests were performed in a conventional three-electrode system on an electrochemical station (CHI 760E) using a SCE electrode as the reference electrode and a graphitic carbon rod as the counter electrode. The electrocatalytic measurements were conducted inside an aqueous solution (50.0 $mmol\cdot L^{-1}$ Na_2SO_4, pH ~ 3.0).

Rotating ring disk electrode (RRDE) characterization. The electrodes were prepared by dispersing the samples in ethanol to achieve a catalyst concentration of ~3.3 $mg\cdot mL^{-1}$ with 5

wt% Nafion. After sonication for 60 min, 6 μL of the catalyst ink was drop-dried onto a glassy carbon disc (area: 0.196 cm^2). The electrochemical tests were performed in a CHI 760E electrochemical workstation with a three-electrode cell at room temperature. The glass carbon electrode loaded with catalyst was used as the working electrode. A graphite rod and a SCE were used as the counter and reference electrode, respectively. The ORR activity and selectivity were investigated by polarization curves and RRDE measurements in O_2-saturated electrolyte at a scan rate of 10 $mV{\cdot}s^{-1}$. Polarization curves in N_2-saturated electrolytes were also recorded as a reference. H_2O_2 selectivity of the CuO and N-CuO on the rotating ring-disk electrode was calculated based on the current of both disc and ring electrodes (Eq. S2). A potential of 1.2 V (versus the reversible hydrogen electrode) was applied on the ring of the working electrode at a speed of 1600 rpm. during the entire testing process.

$$H_2O_2\ \text{yield: } H_2O_2\,(\%) = 200 \times \frac{I_R/N}{I_D + I_R/N} \qquad (2)$$

where I_R is the ring current, I_D is the disk current and N is the collection efficiency (0.37).

Supplementary Figures

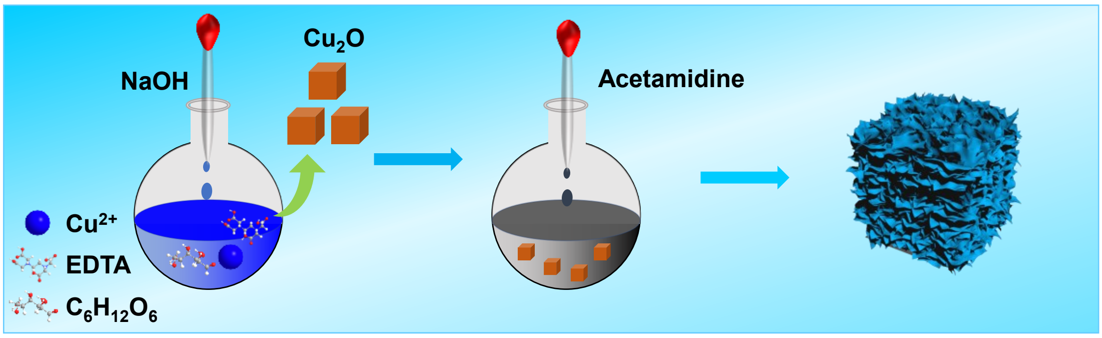

Supplementary Figure 1. Schematic illustration of the N-doping CuO through an acetamidine hydrochloride-assisted ($C_2H_6N_2 \cdot HCl$) oxidation etching strategy.

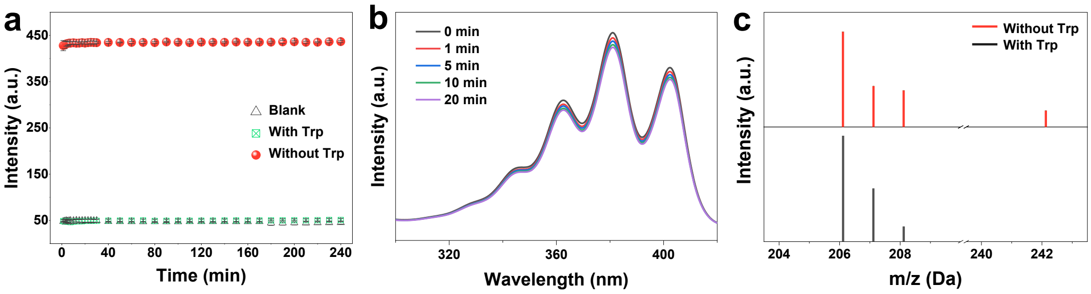

Supplementary Figure 2. a Fluorescence response of SOSG upon treatment in N-CuO system within 240 min with and without the addition of Trp (λ_{ex} = 504 nm and λ_{em} = 525 nm). Error bars represent the standard deviation from triplicate experiments. **b** Excitation and emission spectra of DMA in 10 wt% acetonitrile in H_2O with the addition of Trp in N-CuO system. The excitation was recorded from 280 – 420 nm with λ_{em}= 425 nm. **c** HR-MS chromatogram of the typical DMA and DMA-O_2 from the oxidation of DMA in N-CuO EC system with and without the addition of Trp.

To exclude the contribution of $O_2^{\bullet-}$ for the $^1O_2$ detection, we carried out the control experiments with the addition of Trp (Tryptophan, the quenching agent of $^1O_2$). The results show that the signal of $^1O_2$ is negligible with the addition of Trp, indicating the used chemical approaches for the $^1O_2$ detection are reasonable and can exclude the possible involvement of $O_2^{\bullet-}$.

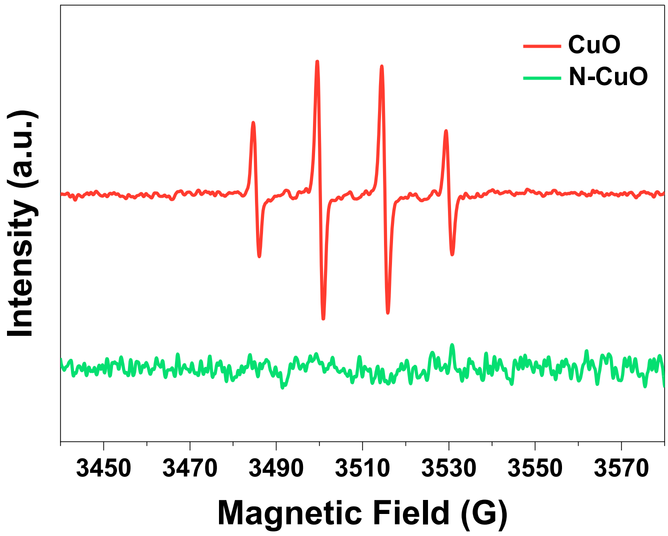

Supplementary Figure 3. EPR spectra for the detection of •OH in the presence of 5,5-dimethyl-1-pyrrolidine-N-oxide (DMPO, 50 mmol·L^{-1}), using water as the solvent.

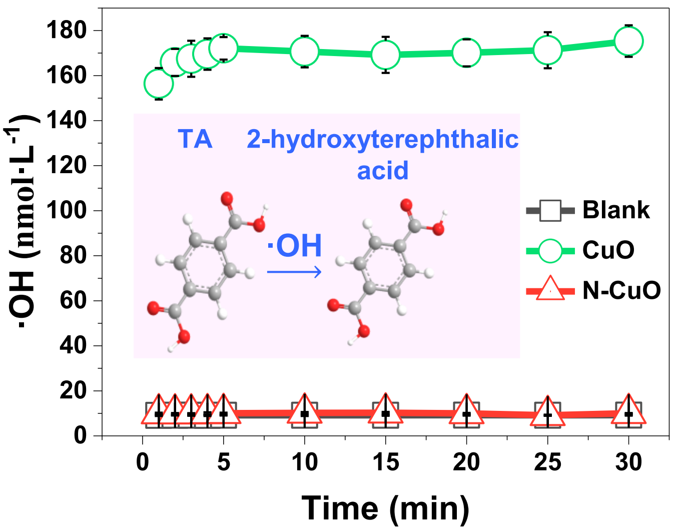

Supplementary Figure 4. The •OH content measured in EC system by TA fluorescence colorimetry. Error bars represent the standard deviation from triplicate experiments.

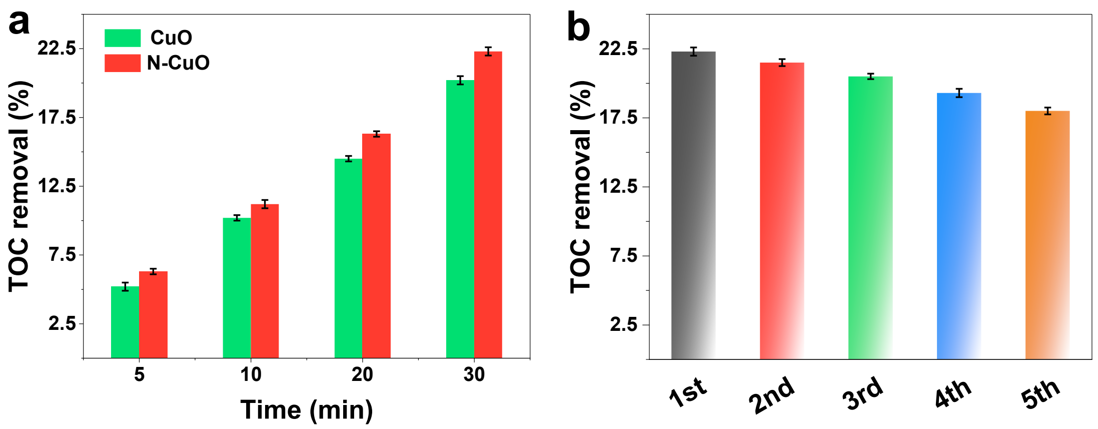

Supplementary Figure 5. a TOC removal of SMX with CuO and N-CuO. **b** Stability test of N-CuO after 5 cycles (The reaction time of each cycle is within 30 min). Error bars represent the standard deviation from triplicate experiments.

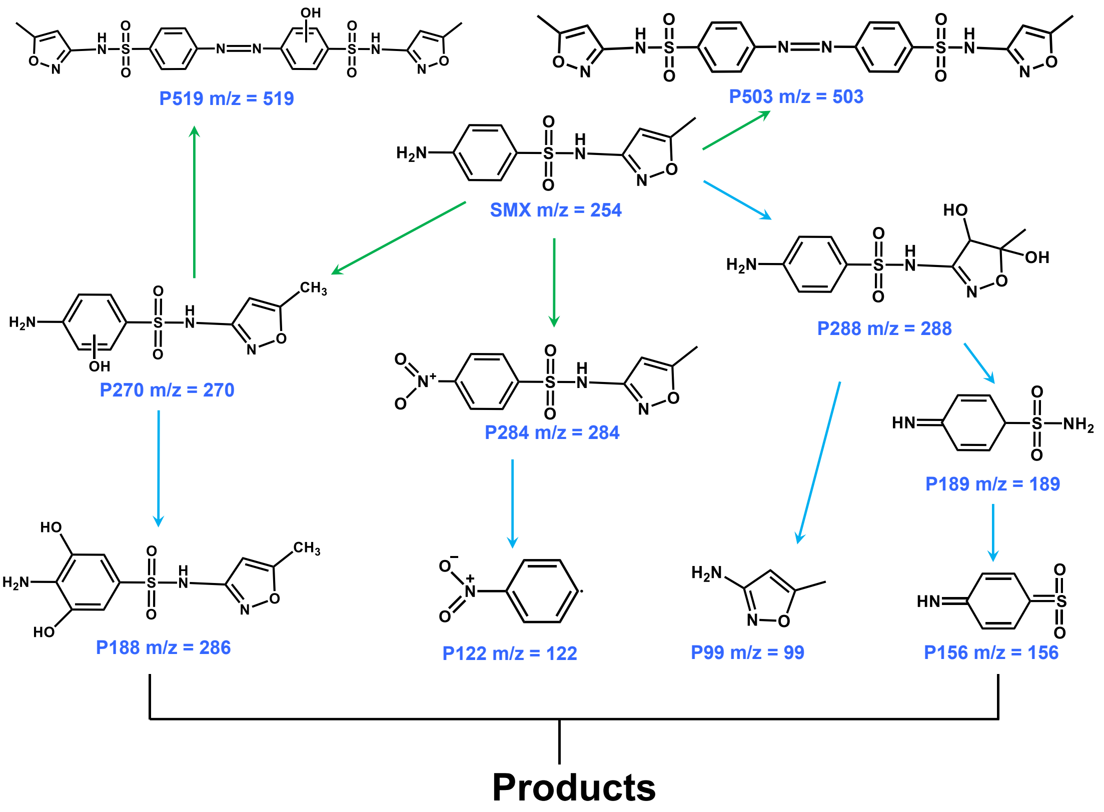

Supplementary Figure 6. Possible degradation pathways of SMX in N-CuO system. The HPLC-MS results confirm the degradation pathways of SMX are consistent with $^1O_2$ oxidation. As expect, the most major ROS for the SMX degradation is $^1O_2$, while $O_2^{\bullet -}$ is believed to be partially involved in the mineralization of reaction intermediates[12-14].

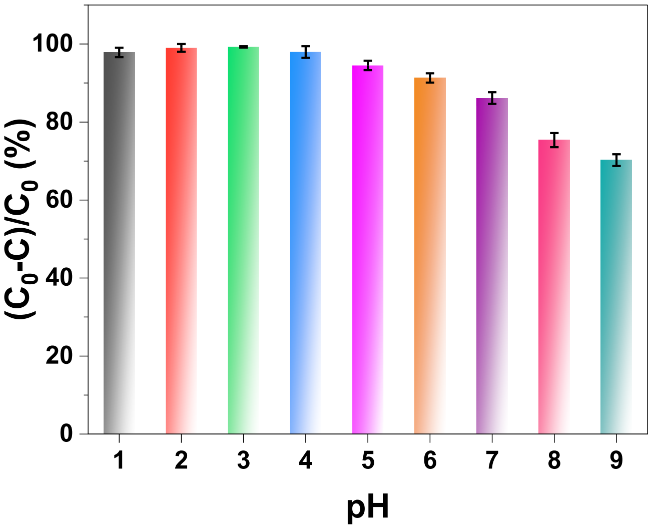

Supplementary Figure 7. Degradation efficiency of SMX in N-CuO system with different pH values. Except for pH values, the reaction conditions were applied potential of 3.0 V, [Na_2SO_4] = 50.0 mmol·L^{-1}, [SMX] = 50.0 mg·L^{-1} and [t] = 30 min. Error bars represent the standard deviation from triplicate experiments.

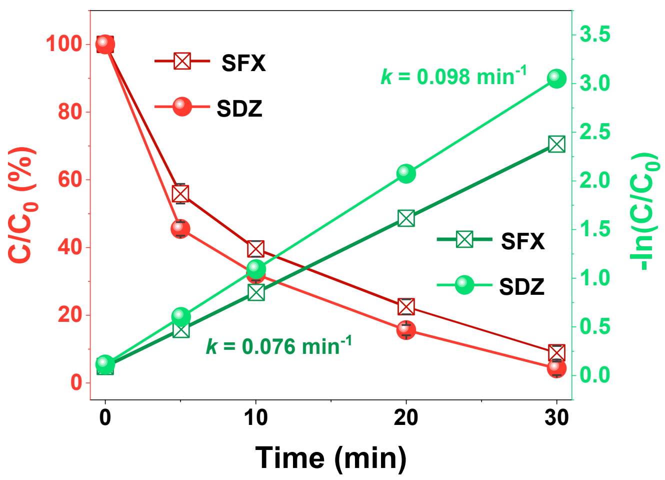

Supplementary Figure 8. The degradation efficiency in N-CuO system toward SFX and SDZ. The reaction conditions were applied potential of 3.0 V, [Na_2SO_4] = 50.0 mmol·L^{-1}, [pollutants] = 50.0 mg·L^{-1} and [t] = 30 min. Error bars represent the standard deviation from triplicate experiments.

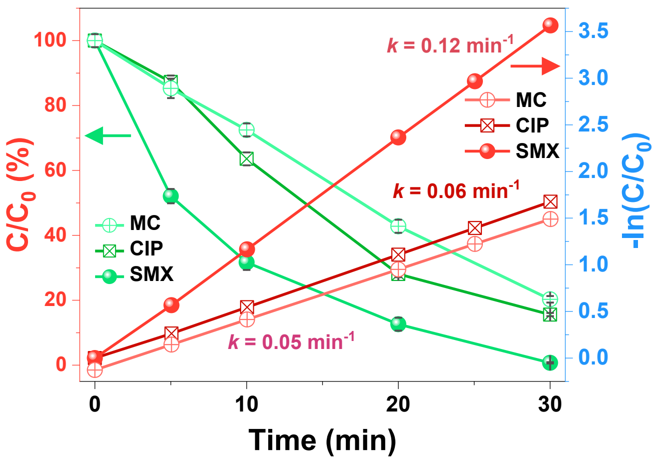

Supplementary Figure 9. The degradation efficiency in N-CuO system toward MC, SMX and CIP. The reaction conditions were applied potential of 3.0 V, [Na_2SO_4] = 50.0 mmol·L^{-1}, [pollutants] = 50.0 mg·L^{-1}. Error bars represent the standard deviation from triplicate experiments.

In light of the selectivity of $^1O_2$ for the removal of electron-rich substances, the removal of the cationic methylrosanilnium chloride (MC) and neutral ciprofloxacin (CIP) was investigated. Results demonstrate that the degradation efficiencies of MC and CIP are only 79.7% and 84.4% after 30 min, and the *k* values are 0.05 min^{-1} and 0.06 min^{-1}, respectively, which mediately confirms $^1O_2$ is the major ROS in N-CuO system and can achieve selective pollutant degradation.

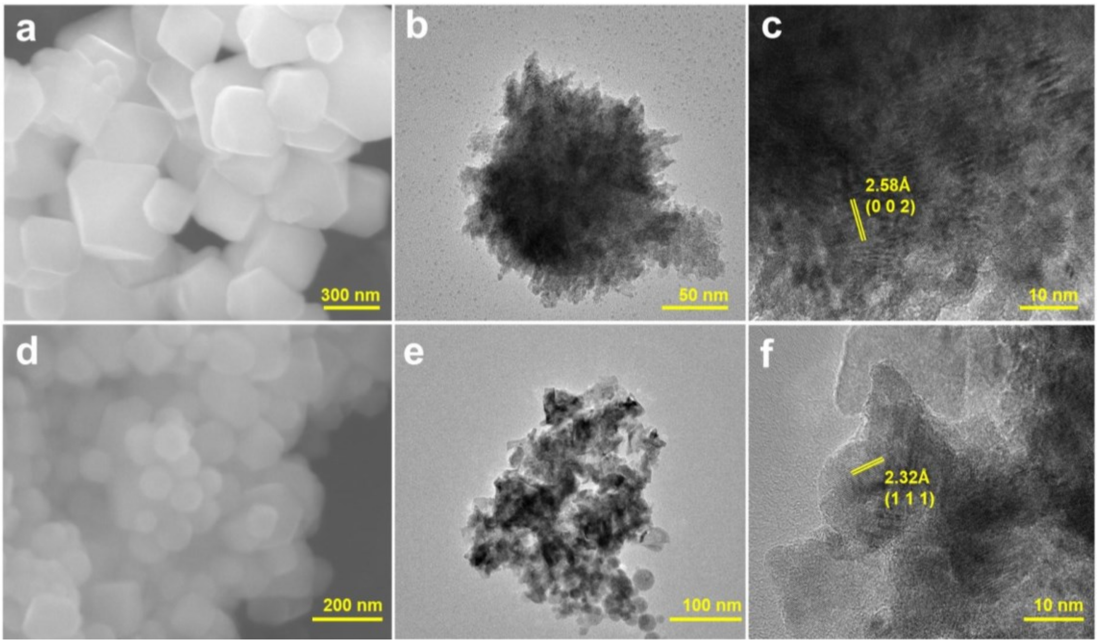

Supplementary Figure 10. a SEM image of OCT-Cu_2O. **b** TEM and **c** HRTEM images of N-CuO-OCT. **d** SEM image of DOD-Cu_2O. **e** TEM and **f** HRTEM images of N-CuO-DOD. Moreover, octahedral Cu_2O (OCT-Cu_2O) and dodecahedra Cu_2O (DOD-Cu_2O) precursors were prepared through the intergrowth method[1]. N-CuO-OCT and N-CuO-DOD were prepared by similar method with N-CuO. From TEM pictures, N-CuO-OCT and N-CuO-DOD exhibit that the interplanar spacings of 2.58 Å and 2.32 Å, which can be indexed as the (002) and (111) *d*-spacing, respectively.

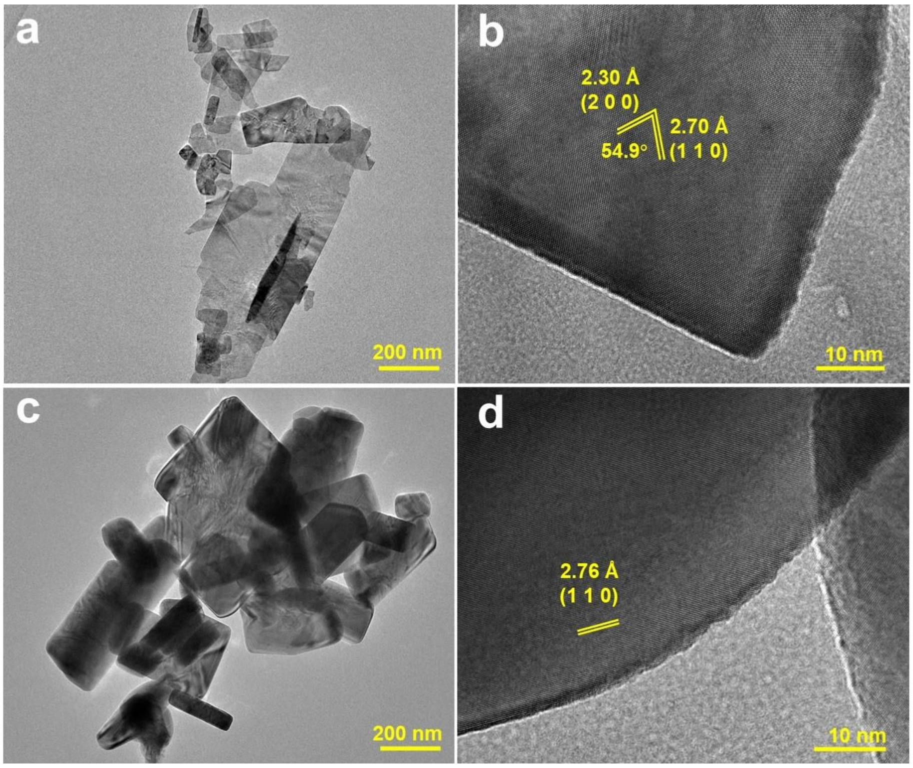

Supplementary Figure 11. **a** TEM and **b** HRTEM images of N-CuO-140. **c** TEM and **d** HRTEM images of N-CuO-200. N-CuO-140 with exposed (200) and (110) lattices as well as N-CuO-200 with exposed (110) lattice were prepared through the hydrothermal method[2,3].

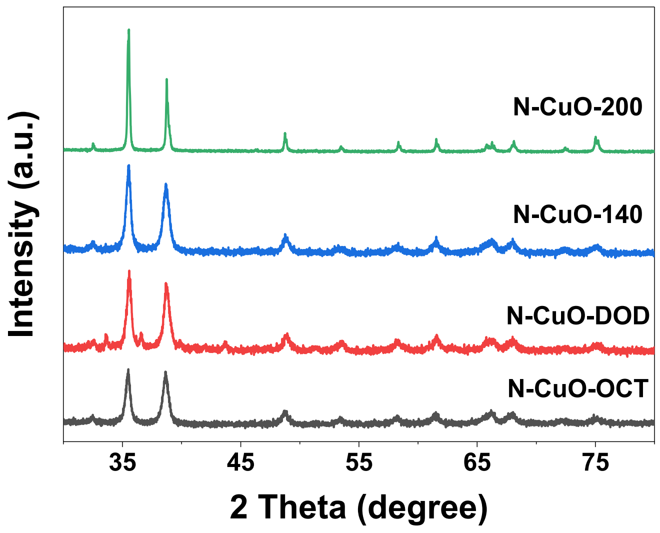

Supplementary Figure 12. XRD patterns of N-CuO-x samples. All the diffraction peaks of N-CuO-x samples can be well indexed as a monoclinic structure without the appearance of any impurity phase.

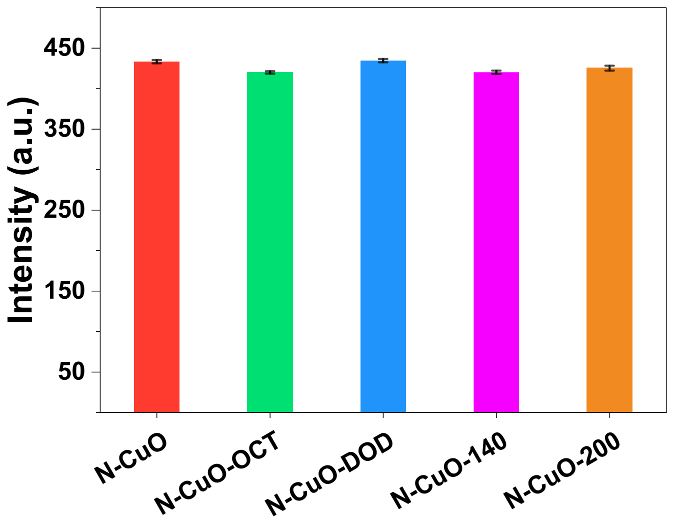

Supplementary Figure 13. Fluorescence response of SOSG upon treatment with N-CuO-x at 15 min (λ_{ex} = 504 nm and λ_{em} = 525 nm). Error bars represent the standard deviation from triplicate experiments. The results show that no significant difference can be observed for N-CuO-x samples with different exposed lattices, indicating N-doping effect accounts for the higher catalytic performance of N-CuO than that of facet effect.

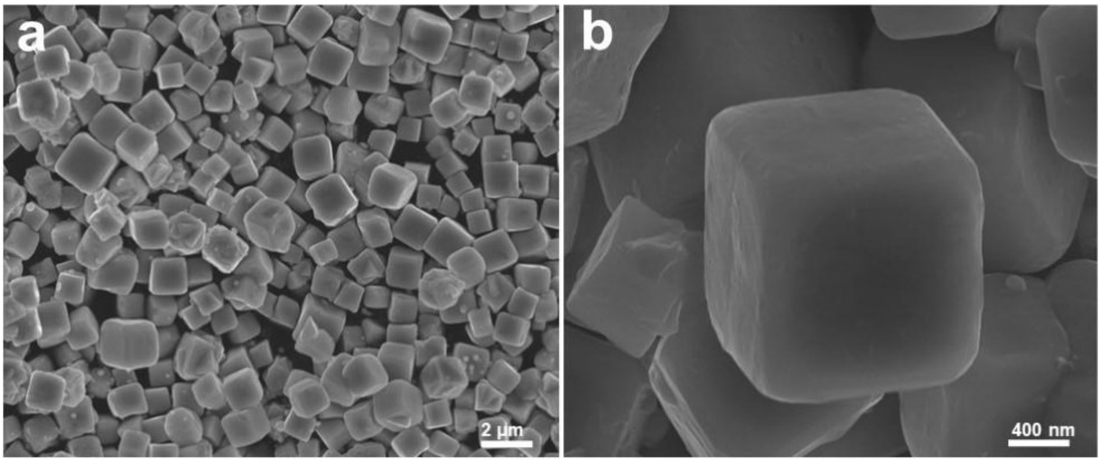

Supplementary Figure 14. SEM images of Cu_2O precursor.

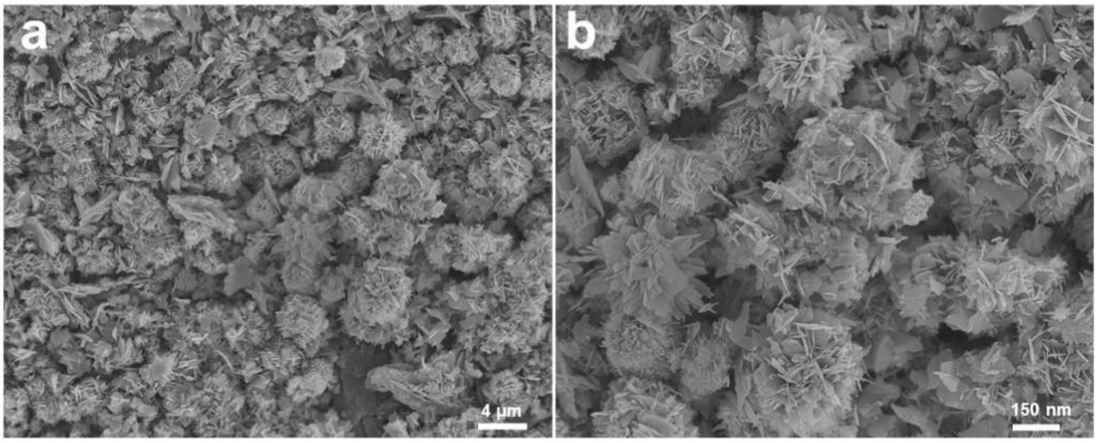

Supplementary Figure 15. SEM images of N-CuO hierarchical cube.

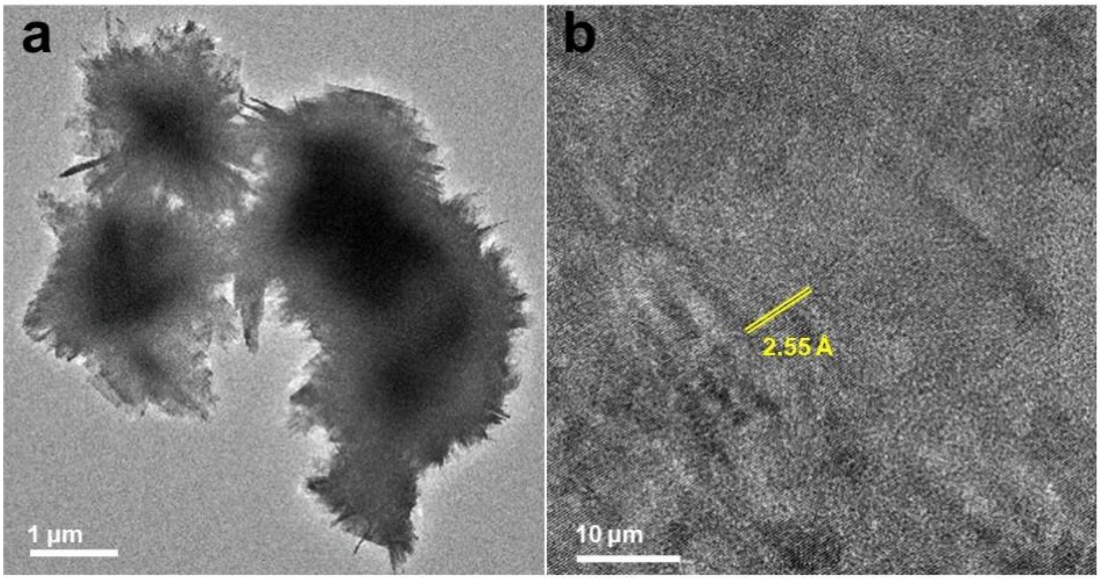

Supplementary Figure 16. TEM images of N-CuO hierarchical cube.

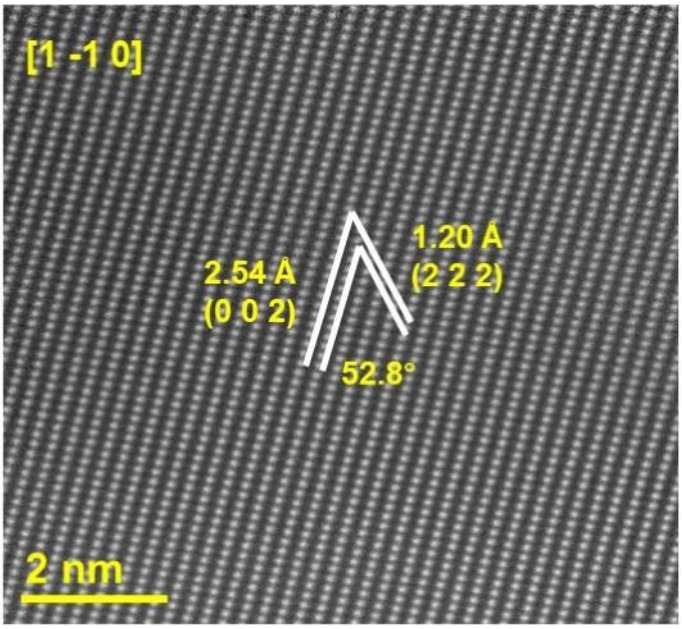

Supplementary Figure 17. The HAADF-STEM image of CuO. The HAADF signal intensity is proportional to the square of the atomic number. Thus, the atomic columns containing Cu atoms are bright, whereas the lighter O and/or N atoms are not observable.

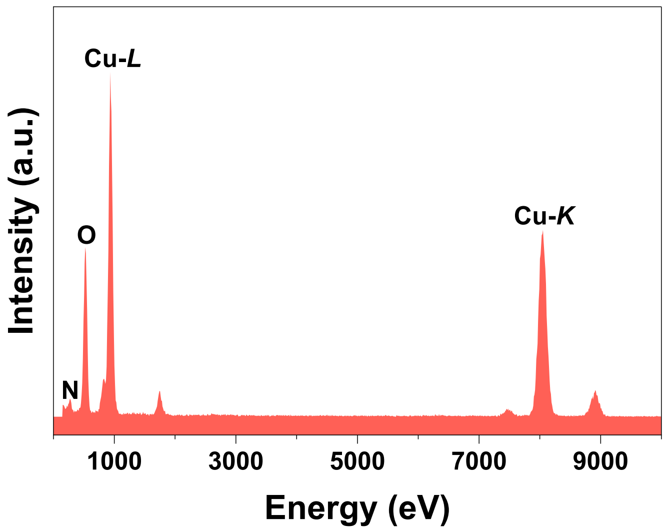

Supplementary Figure 18. The EDX spectrum of N-CuO hierarchical cube.

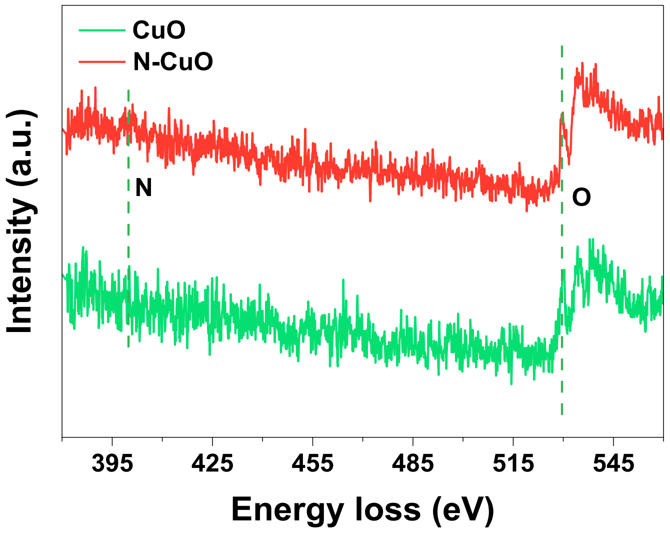

Supplementary Figure 19. O and N *L*-edge EELS spectra of CuO and N-CuO. The N *L*-edge is less pronounced due to the low N content in the N-CuO. EELS shift of O element to higher energy indicates that the N-CuO with higher oxidation states because of the electron transfer from O to N[15].

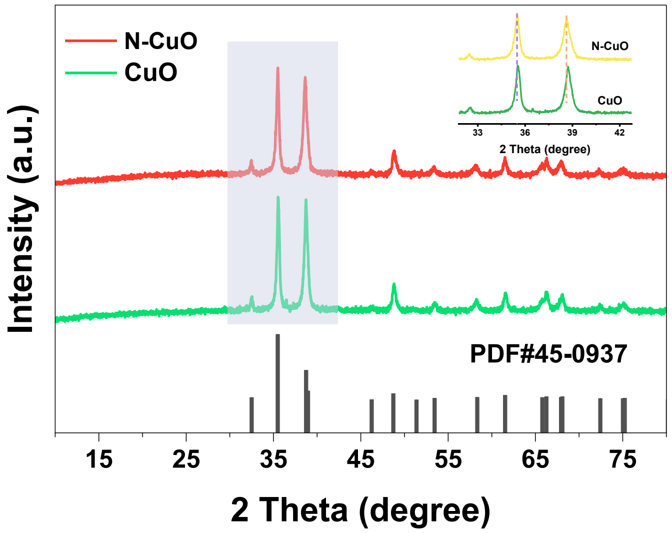

Supplementary Figure 20. XRD patterns of CuO and N-CuO. All the diffraction peaks of samples can be well indexed as a monoclinic structure without the appearance of any impurity phase. Notably, the main peaks of N-CuO shift slightly to lower angles compared to the pristine CuO, implying a lattice distortion after N doping, which is in conformity to the HAADF-TEM result.

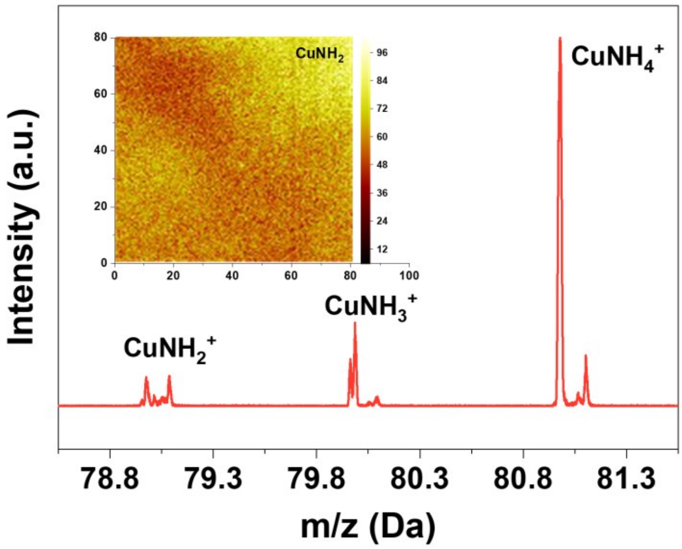

Supplementary Figure 21. TOF-SIMS of N-CuO (The inset is the mapping of TOF-SIMS).

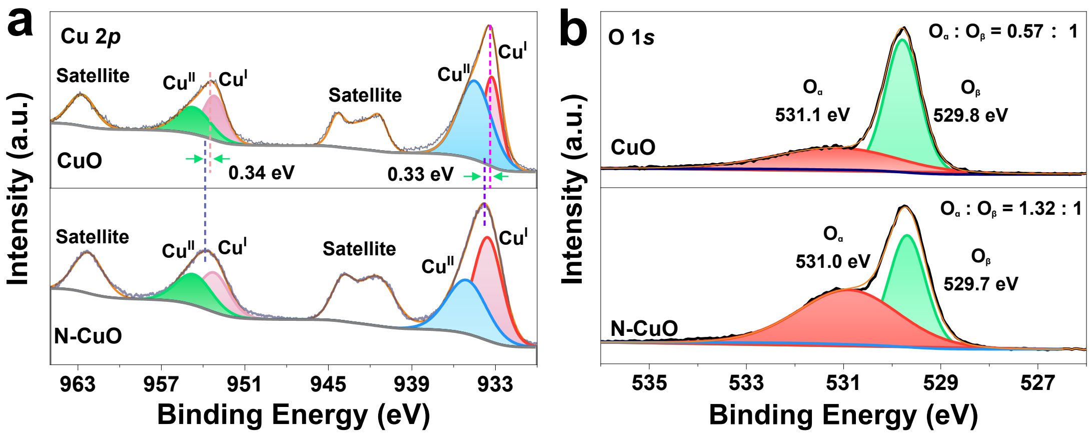

Supplementary Figure 22. a, b XPS spectra of the Cu 2*p* (**a**) and O 1*s* (**b**) on CuO and N-CuO. Shifts of Cu^{+}/Cu^{2+} peaks of N-CuO toward higher binding energy is observed as compared to those of bare CuO, which are about 0.34 eV and 0.33 eV, respectively, indicating that Cu have been partial oxidized. This confirms the strong electron interactions due to the N dopant in the N-CuO, which may change the electron transport properties of catalysts, and thus benefits the EC activity[15].

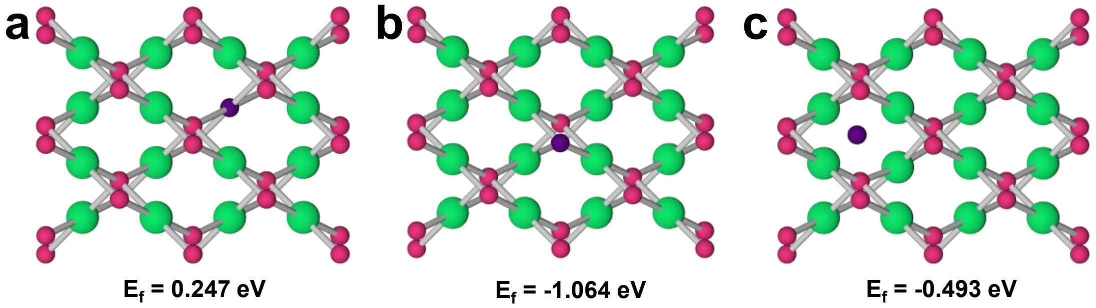

Supplementary Figure 23. The surface structures of N doped CuO (222). It shows that interstitial N and substitutional N in the Cu sites have higher energy, indicating the most stable structure is the N atoms substitute for the surface O atoms.

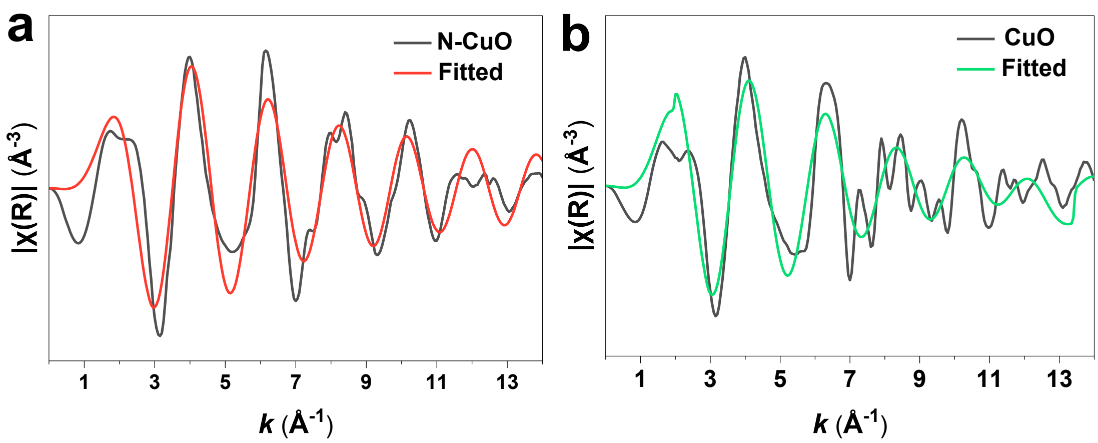

Supplementary Figure 24. a, b The corresponding EXAFS fitting curves of (**a**) N-CuO and (**b**) CuO at *k* space, respectively.

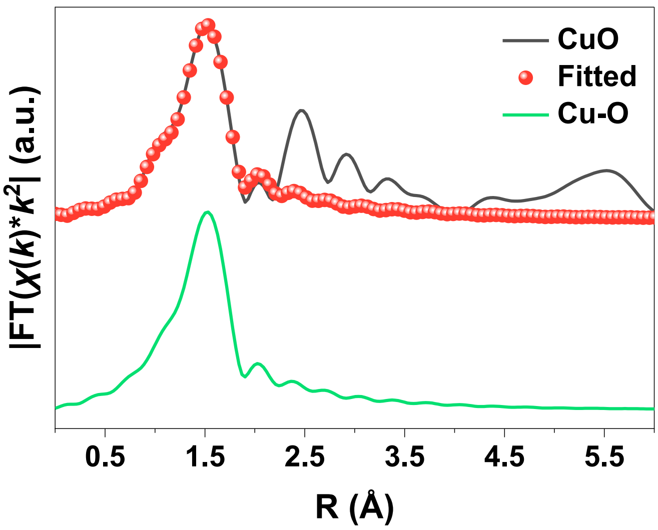

Supplementary Figure 25. EXAFS analysis of CuO at R space.

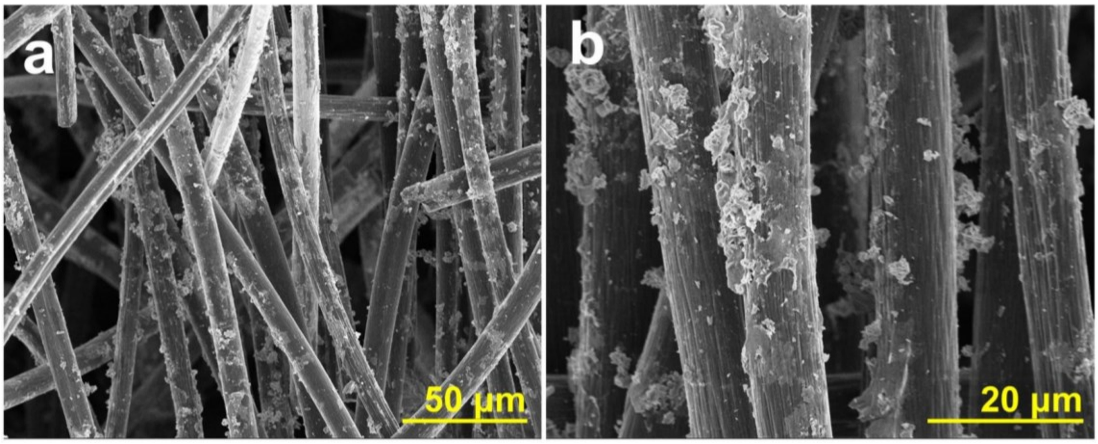

Supplementary Figure 26. a, b SEM images of CF electrode loaded N-CuO. The SEM images of carbon felt (CF) loaded N-CuO show that carbon fibers interlace with each other to form a complex three-dimensional network structure. Also, the catalysts are evenly coated with N-CuO particles.

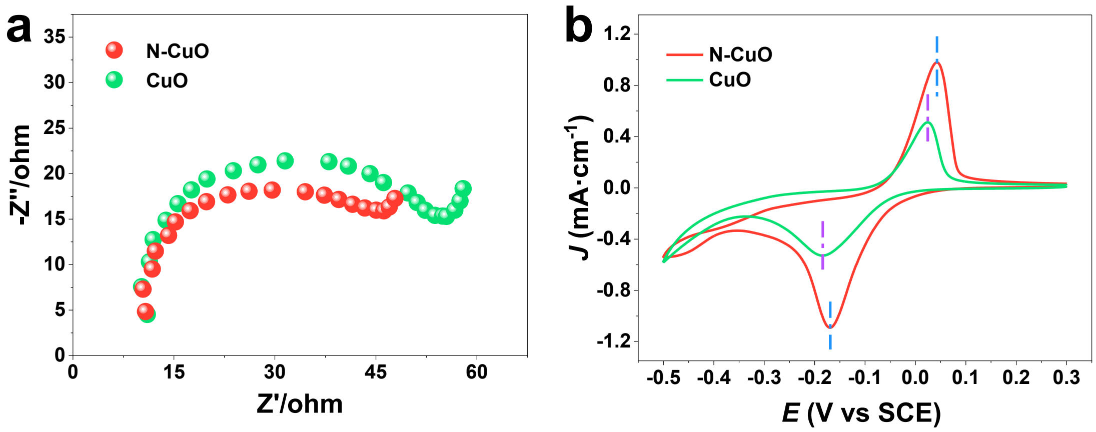

Supplementary Figure 27. a Nyquist plots of samples under a potential of 0.2 V in Na_2SO_4 aqueous solution (50.0 mmol·L^{-1} Na_2SO_4 and pH ~ 3.0). **b** Cyclic voltammogram of electrodes in Na_2SO_4 aqueous solution (50.0 mmol·L^{-1} Na_2SO_4 and pH ~ 3.0). All potentials were referenced to saturated calomel electrode (SCE). All the scan rates were 50 mV·s^{-1} and were performed from the negative potential to positive potential.

The electrochemical behavior of electrodes was investigated by electrochemical impedance spectra (EIS) and cyclic voltammetry (CV). Supplementary Fig. 27a exhibits the Nyquist plots for CuO and N-CuO electrodes. Also, the charge transfer resistance on the electrode surface can be represented by the effective diameter of a semicircle. The radius of the semicircle of N-CuO is smaller than that of CuO, which indicates that the composite electrodes had better charge transfer ability due to the doping of N. From Supplementary Fig. 27b, it is found that the redox peak current and peak area values of the N-CuO composite increased compared with CuO, which is caused by the doping of N.

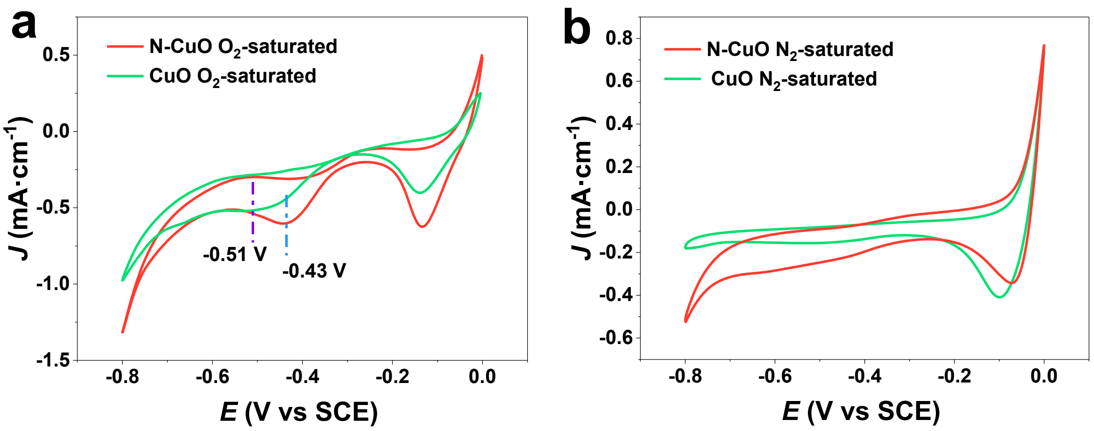

Supplementary Figure 28. a, b Cyclic voltammogram of CuO and N-CuO in (**a**) O_2- and (**b**) N_2-saturated 50 mmol·L^{-1} Na_2SO_4 (pH = 3) aqueous solution. All potentials were referenced to saturated calomel electrode (SCE). All the scan rates were 50 mV·s^{-1} and were performed from the negative potential to positive potential.

The oxygen reduction activity of CuO and N-CuO were examined by cyclic voltammetry (CV) in N_2- and O_2-saturated solution. As shown in Supplementary Fig. 28a, b for all electrocatalysts, reduction peaks appear in the CV curves measured in O_2-saturated solution, whereas CV curves without any noticeable peaks are obtained in N_2-saturated solution, indicating the electrocatalytic oxygen reduction occurs in the N-CuO and CuO systems. Furthermore, reduction peaks of CuO and N-CuO appear different, that elucidate CuO with and without N dopant have different electrochemical behavior for the molecular oxygen activation process.

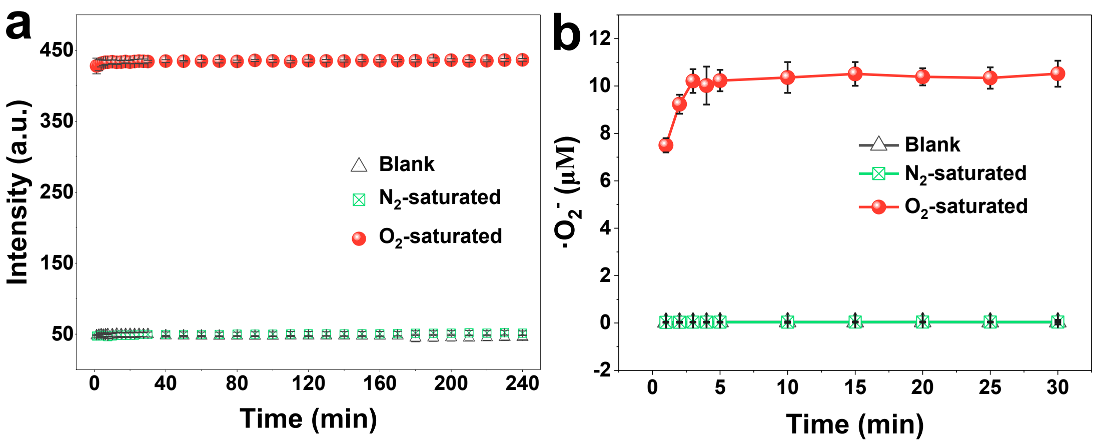

Supplementary Figure 29. a Fluorescence response of SOSG upon treatment in N-CuO system within 240 min under the O_2 and N_2-saturated atmosphere (λ_{ex} = 504 nm and λ_{em} = 525 nm). **b** The amount of •OOH/$O_2^{\bullet-}$ in N-CuO system under the N_2- and O_2-saturated atmosphere. Error bars represent the standard deviation from triplicate experiments.

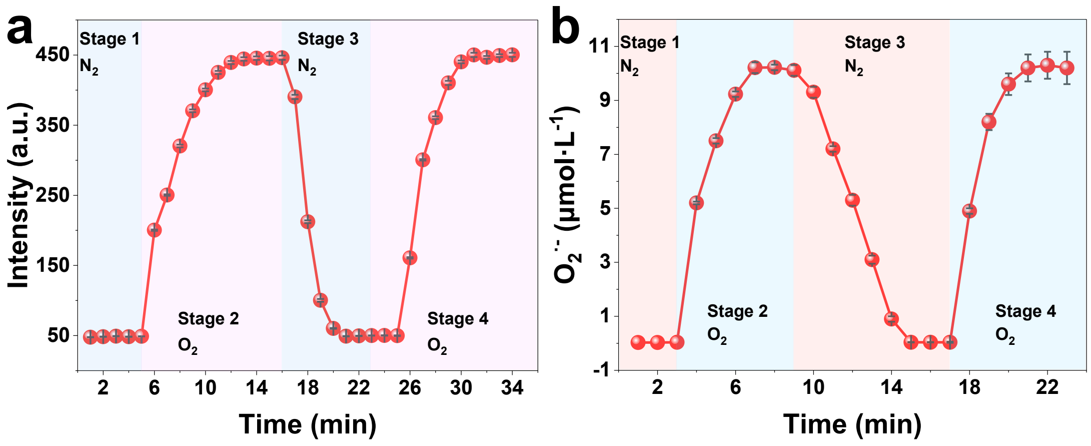

Supplementary Figure 30. a Fluorescence response of SOSG and **b** the amount of •OOH/$O_2^{•-}$ upon treatment in N-CuO system under the intermittent N_2 and O_2 aeration. Error bars represent the standard deviation from triplicate experiments.

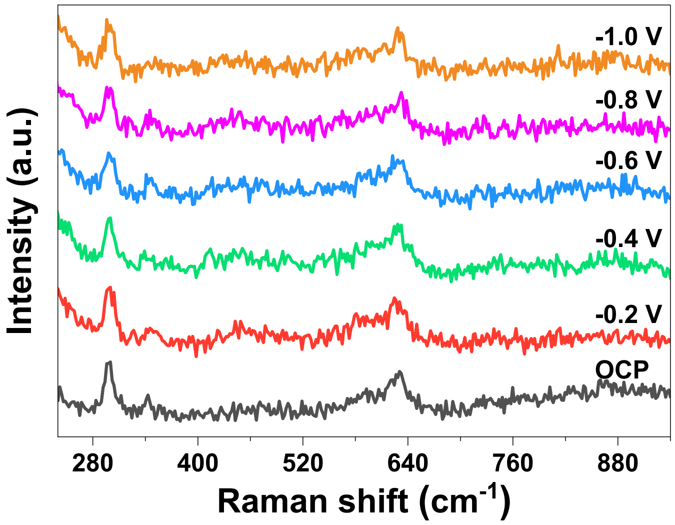

Supplementary Figure 31. In situ Raman spectra of N-CuO in EC system under N_2 atmosphere.

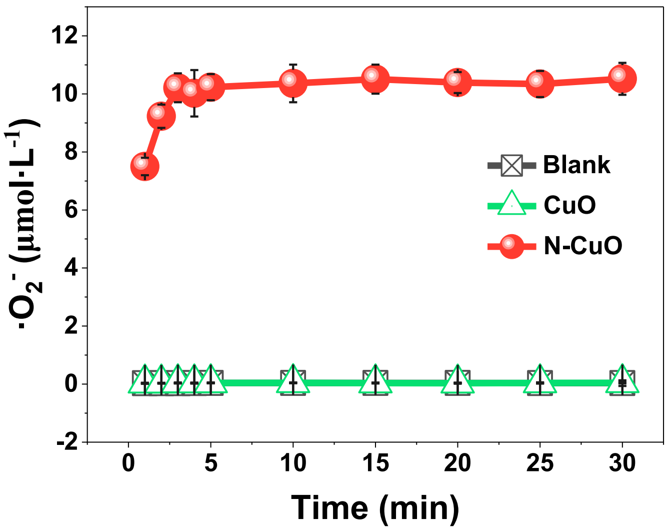

Supplementary Figure 32. The amount of •OOH/$O_2^{\bullet-}$ in EC system. Error bars represent the standard deviation from triplicate experiments.

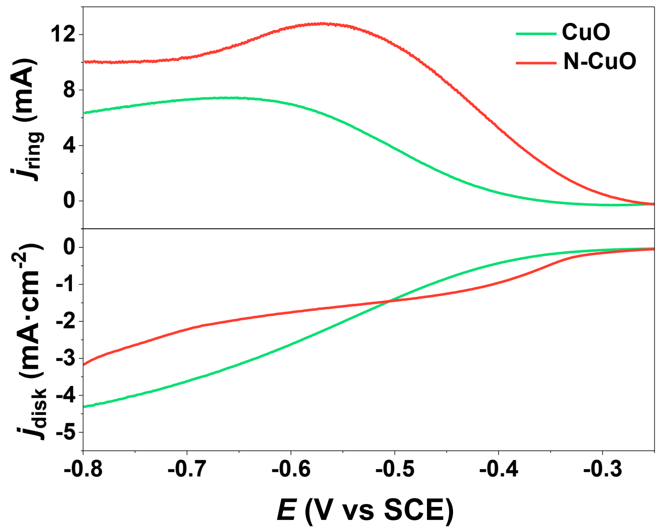

Supplementary Figure 33. RRDE measurement of the selective oxygen reduction of catalysts in an aqueous solution (50.0 mmol·L^{-1} Na_2SO_4 and pH ~ 3.0). The decrease of limited diffusion current density (j_{lim}) for N-CuO is explained in terms of the desorption for •OOH intermediate and its necessary diffusion to the bulk solution, with the subsequent loss of Faradaic efficiency[16].

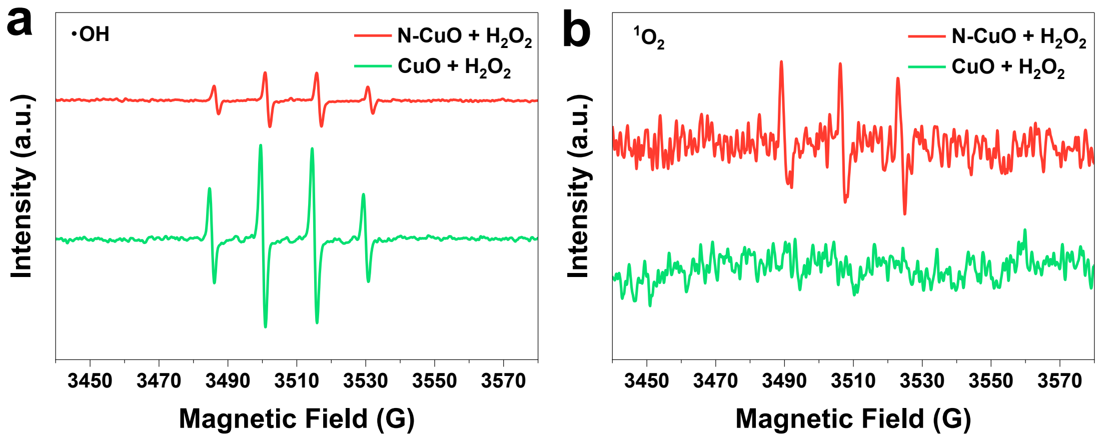

Supplementary Figure 34. EPR spectra in CuO and N-CuO systems with the addition of H_2O_2.

To further verify the source of $^1O_2$ and the effect of H_2O_2, we carried out the control experiments with the addition of H_2O_2. It could be seen that when the existence of H_2O_2, the •OH will be produced inevitably, confirming that the N-CuO in EC system is mainly selective generation of $^1O_2$.

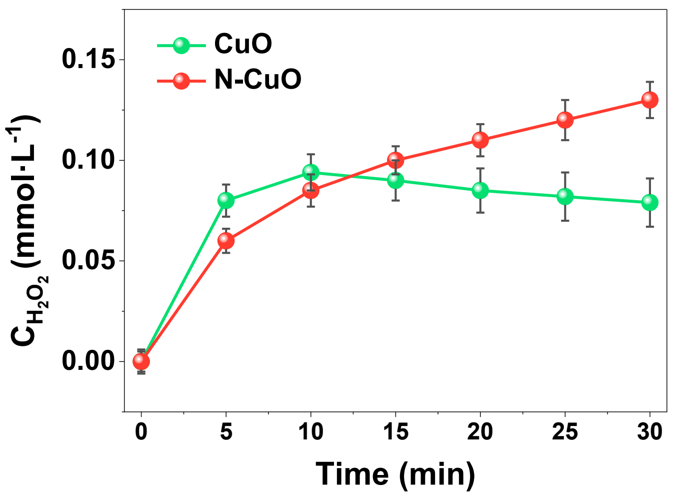

Supplementary Figure 35. The concentrations of H_2O_2 in N-CuO and CuO systems. Error bars represent the standard deviation from triplicate experiments.

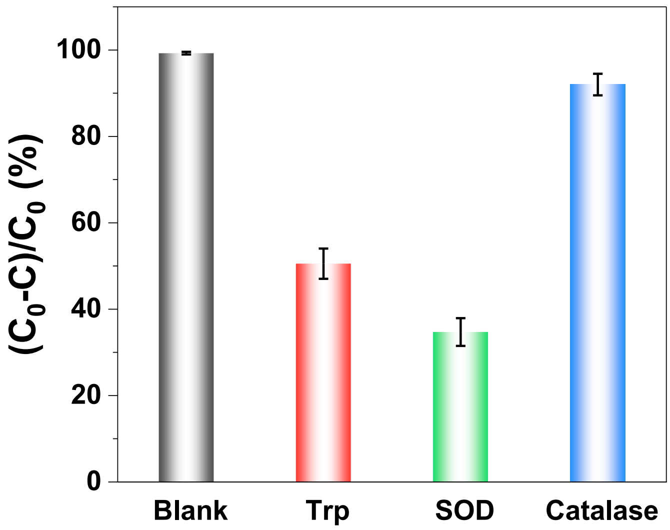

Supplementary Figure 36. ROS quenching by applying 10.0 mmol·L^{-1} SOD, 2.0 mg·L^{-1} catalase, and 10.0 mmol·L^{-1} Trp as quenchers for $O_2^{\bullet -}$, H_2O_2, and $^1O_2$, respectively. Except for different sacrificial agents, the reaction conditions were applied potential of 3.0 V, [Na_2SO_4] = 50.0 mmol·L^{-1}, [SMX] = 50.0 mg·L^{-1} and [t] = 30 min. Error bars represent the standard deviation from triplicate experiments.

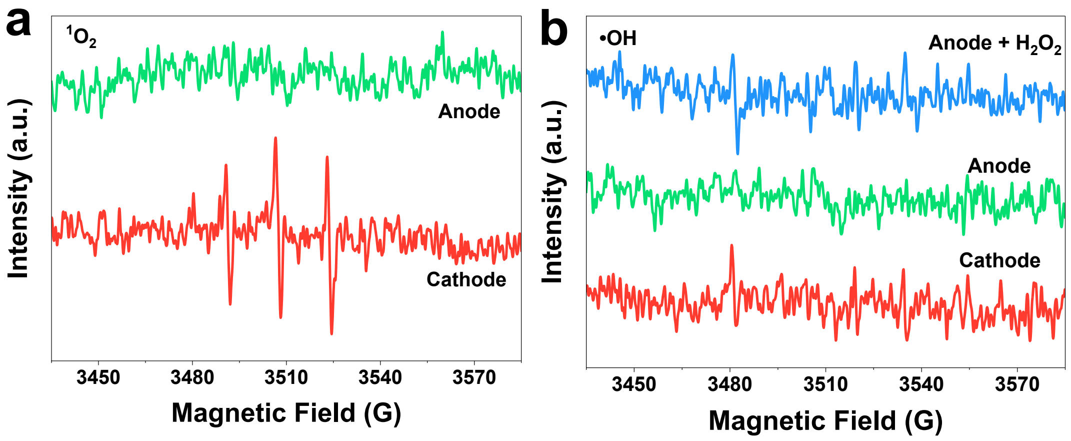

Supplementary Figure 37. a,b The ROS generation occurs at the anode and cathode in EC system, EPR spectra of (**a**) $^1O_2$ and (**b**) •OH. In order to distinguish the sources of $^1O_2$, we employed H-type electrolytic cell, in which the anode and cathode sides were separated by a Nafion 117 membrane, to monitor the cathodic and anodic reactions. Results show that the $^1O_2$ is coming from the cathodic reaction as there is almost no $^1O_2$ signal at the anode. Furthermore, the other ROS is not detected near the anode, which can exclude the effort of anodic reaction of $^1O_2$ generation.

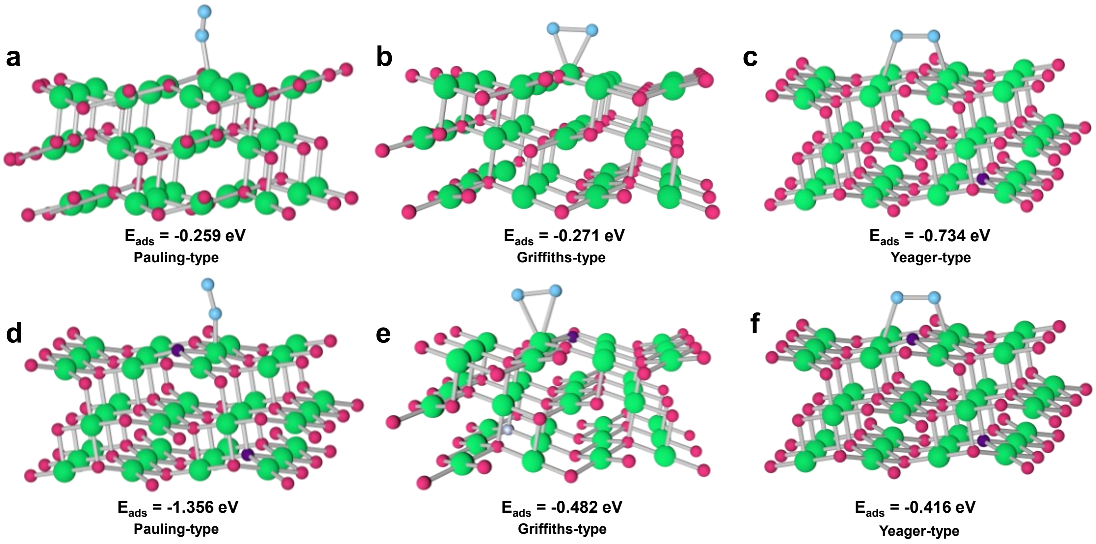

Supplementary Figure 38. The adsorption configurations of oxygen on the surfaces of CuO (222) (**a-c**) and N-CuO (222) (**d-f**).

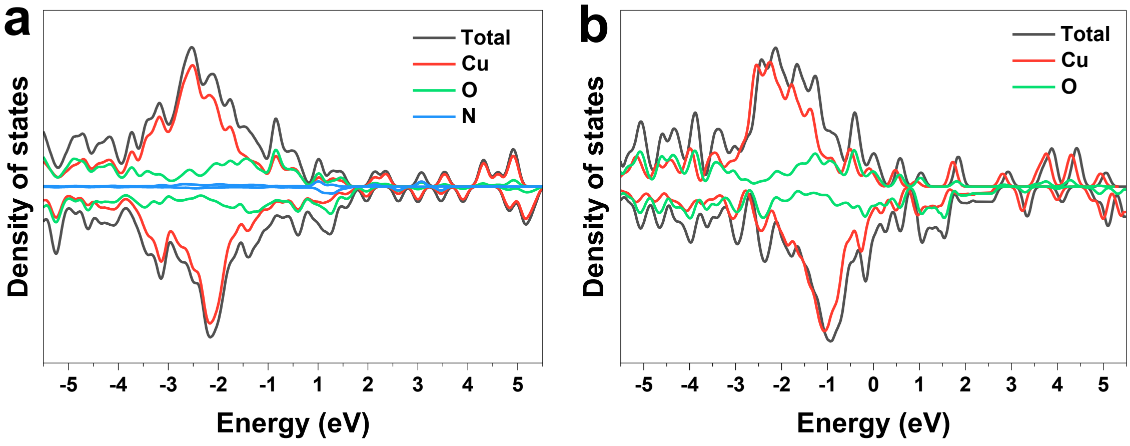

Supplementary Figure 39. a, b Density of states of (**a**) N-CuO (222) and (**b**) CuO (222).

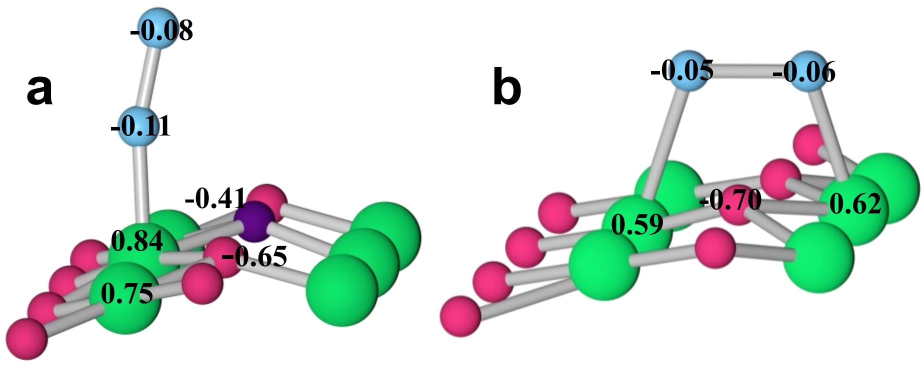

Supplementary Figure 40. a, b Bader charge analysis of catalysts for (**a**) N-CuO and (**b**) CuO after O_2 adsorption.

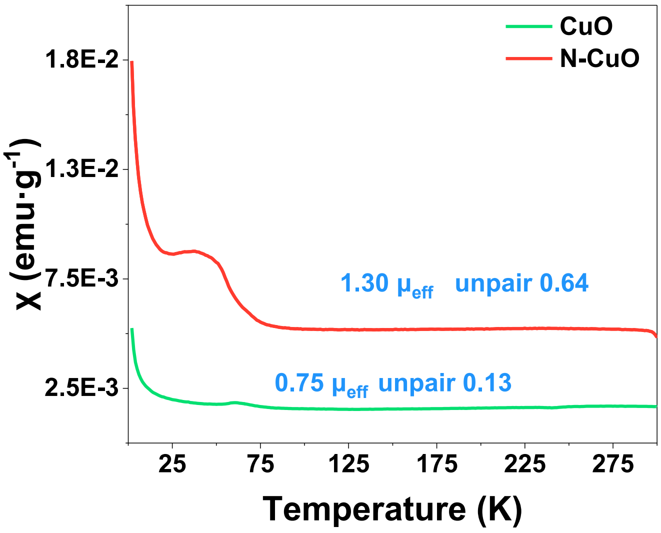

Supplementary Figure 41. Temperature-dependent magnetization characterizations of the as-prepared samples.

Supplementary Tables

Supplementary Table 1. Comparison of the catalytic activities of N-CuO with the other catalysts in the Literature

Catalyst ($g \cdot L^{-1}$)	System	Pollutant ($mg \cdot L^{-1}$)	T (°C)	t (min)	TOF (min^{-1})	Efficiency (%)	Ref.
Co^{2+}@PMAP (0.4)	PMS Fenton-like	Phenol (10)	25	50	0.185	100	10
MgO (0.5)	PMS Fenton-like	BPA (5.0)	25	40	0.011	100	11
NC-ZiF-8 (0.2)	PMS Fenton-like	Phenol (20)	25	60	0.39	100	12
1.0/1 Ca-BSACs (0.2)	PMS Fenton-like	BPA (22.83)	20	50	8.02×10^{-4}	100	13
Cu-Al_2O_3 (1.0)	H_2O_2 Fenton-like	BPA (20)	25	180	0.01	87	14
Fe-$Ce_{0.75}Zr_{0.25}O_2$ (80)	Electro-Fenton	PAM (200)	25	120	2.32×10^{-11}	86	15
N-CuO (0.077)	Electrocatalysis	SMX (50)	25	30	0.41	99	This work

Supplementary Table 2. EXAFS fitting parameters at the Cu K-edge for various samples

Sample	Shell	N [a]	R (Å) [b]	σ^2 (Å^2·10^{-3}) [c]	ΔE_0 (eV) [d]	R factor (%)
N-CuO	Cu-N	1	2.04401	2.64	3.880	0.52
	Cu-O	3	1.91493	1.91	-1.704	
CuO	Cu-O	3.9	1.96813	0.51	-0.214	1.2

Supplementary Notes

Supplementary Note 1

As the reason that proton affects the $^1O_2$ generation due to pK_a of $HO_2^{\bullet-}$. The dynamics for the hydrolysis and disproportionation of superoxide ($O_2^{\bullet-}$) in aqueous media have been characterized in previous work by pulse radiolysis[17]. According to the acid-base dissolution equilibrium theory, the formation of $HO_2^{\bullet-}$ decreases with the decrease of hydrogen ion concentration.

$$HO_2^{\bullet-} \rightleftharpoons O_2^{\bullet-} + H^+ \quad (pK_a = 4.8) \tag{3}$$

$$HO_2^{\bullet-} + O_2^{\bullet-} \rightarrow H_2O_2 + {}^1O_2 \qquad k_1 = 1.0 \times 10^8\ mol^{-1}\cdot s^{-1} \tag{4}$$

$$O_2^{\bullet-} + O_2^{\bullet-} + 2H_2O \rightarrow H_2O_2 + {}^1O_2 + 2OH^- \qquad k_2 < 0.3\ mol^{-1}\cdot s^{-1} \tag{5}$$

Furthermore, the kinetic constants of $^1O_2$ generation involving $O_2^{\bullet-}$ and $HO_2^{\bullet-}$ reveal that the formation of $HO_2^{\bullet-}$ can improve the $^1O_2$ generation kinetic due to the fastest reaction kinetic constant for the $^1O_2$ generation (Eq. S3-S5)[1-3].

When the hydrogen ion concentration in the solution is high (that is the lower pH value), it is beneficial to the accumulation of $HO_2^{\bullet-}$ in the solution. Since $HO_2^{\bullet-}$ is an important intermediate for the $^1O_2$ generation, the pH value will affect the degradation kinetics of SMX.

Supplementary Note 2

The strong Pauling-type adsorption of O_2 at N-CuO can be attributed to the density of state (DOS) at the Fermi level, then more energy levels appear in the conduction band of N-CuO, and the number of Fermi level electrons increases. Therefore, the introduction of N results in charge deletion on the Cu centers around the N coordination; hence, these charge-deletion Cu in the N-CuO could enhance oxygen adsorption, electron conduction and accelerates the reaction rate, which could more effectively stabilize the rate-limiting *OOH intermediates compared to the Cu sites coordinated with four oxygen atoms in the pristine CuO, finally promoting the performance of $^1O_2$ production.

Supplementary Note 3

To further unravel the electron configuration of N-CuO, the zero-field cooling (ZFC) temperature-dependent magnetic susceptibility was measured[18]. The calculated effective magnetic moments of CuO and N-CuO are 0.75 μ_{eff} and 1.30 μ_{eff}, respectively. Besides, we further obtained the number of unpaired d electron (n) of Cu^{2+} ion by the following equation:

$$\mu_{eff} = \sqrt{n(n+2)} \tag{6}$$

whereby the number of unpaired *d* electron (n) of N-CuO is about 0.64, which means Cu^{2+} ions have a lower electron filling, so that N-CuO leads to a very strong Cu/O_2 interaction. While the number of unpaired d electron (n) of CuO is about 0.13. Certainly, the N doping permits N-CuO to achieve the ideal O_2 adsorption. It is reckoned that this intrinsically optimal electronic configuration would endow the as-designed N-CuO with a high catalytic activity.

References

1. Zhan, C., *et al.* Critical Roles of Doping Cl on Cu_2O Nanocrystals for direct epoxidation of propylene by molecular oxygen. *J. Am. Chem. Soc.* **142**, 14134-14141 (2020).
2. Liu, X., *et al.* Morphology- and facet-controlled synthesis of CuO micro/nanomaterials and analysis of their lithium ion storage properties. *J. Power Sources* **312**, 199-206 (2016).
3. Xiao, B., Wu, M., Wang, Y., Chen, R. & Liu, H. Sulfite activation and tetracycline removal by rectangular copper oxide nanosheets with dominantly exposed (001) reactive facets: Performance, degradation pathway and mechanism. *Chem. Eng. J.* **406**, 126693 (2021).
4. Dou, X., *et al.* MoS_2-quantum dot triggered reactive oxygen species generation and depletion: responsible for enhanced chemiluminescence. *Chem. Sci.* **10**, 497-500 (2019).
5. Reybier, K., *et al.* Free superoxide is an intermediate in the production of H_2O_2 by copper(I)-Aβ peptide and O_2. *Angew. Chem.* **128**, 1097-1101 (2016).
6. Ye, L., Liu, J., Jiang, Z., Peng, T. & Zan, L. Facets coupling of BiOBr-g-C_3N_4 composite photocatalyst for enhanced visible-light-driven photocatalytic activity. *Appl. Catal. B: Environ.* **142-143**, 1-7 (2013).
7. Hirakawa, T., Yawata, K. & Nosaka, Y. Photocatalytic reactivity for $O_2{\bullet}^-$ and •OH radical formation in anatase and rutile TiO_2 suspension as the effect of H_2O_2 addition. *Appl. Catal. A: Gen.* **325**, 105-111 (2007).
8. Freinbichler, W., *et al.* Validation of a robust and sensitive method for detecting hydroxyl radical formation together with evoked neurotransmitter release in brain microdialysis. *J. Neurochem.* **105**, 738-749 (2008).
9. Mahne, N., *et al.* Singlet oxygen generation as a major cause for parasitic reactions during cycling of aprotic lithium-oxygen batteries. *Nat. Energy* **2**, 17036 (2017).
10. Lu, Z., *et al.* High-efficiency oxygen reduction to hydrogen peroxide catalysed by oxidized carbon materials. *Nat. Catal.* **1**, 156-162 (2018).
11. Zhao, Y., *et al.* Janus electrocatalytic flow-through membrane enables highly selective singlet oxygen production. *Nat. Commun.* **11**, 6228 (2020).
12. Yan, J., *et al.* Activation $CuFe_2O_4$ by hydroxylamine for oxidation of antibiotic sulfamethoxazole. *Environ. Sci. Technol.* **52**, 14302-14310 (2018).

13. Ding, S., Niu, J., Bao, Y. & Hu, L. Evidence of superoxide radical contribution to demineralization of sulfamethoxazole by visible-light-driven $Bi_2O_3/Bi_2O_2CO_3/Sr_6Bi_2O_9$ photocatalyst. *J. Hazard. Mater.* **262**, 812-818 (2013).

14. Zou, Y., Qi, H. & Sun, Z. In-situ catalytic degradation of sulfamethoxazole with efficient CuCo-O@CNTs/NF cathode in a neutral electro-Fenton-like system. *Chemosphere* **296**, 134072 (2022).

15. Zheng, Y.-R.*, et al.* Doping-induced structural phase transition in cobalt diselenide enables enhanced hydrogen evolution catalysis. *Nat. Commun.* **9**, 2533 (2018).

16. Briega-Martos, V., Cheuquepán, W. & Feliu, J.M. Detection of superoxide anion oxygen reduction reaction intermediate on Pt(111) by infrared reflection absorption spectroscopy in neutral pH conditions. *J. Phys. Chem. Lett.* **12**, 1588-1592 (2021).

17. Chin, D.H., Chiericato Jr, G., Nanni Jr, E.J. & Sawyer, D.T. Proton-induced disproportionation of superoxide ion in aprotic media. *J. Am. Chem. Soc.* **104**, 1296-1299 (1982).

18. Yang, G.*, et al.* Regulating Fe-spin state by atomically dispersed Mn-N in Fe-N-C catalysts with high oxygen reduction activity. *Nat. Commun.* **12**, 1734 (2021).
